# The Construction and Use of Log-Odds Substitution Scores for Multiple Sequence Alignment

**DOI:** 10.1371/journal.pcbi.1000852

**Published:** 2010-07-15

**Authors:** Stephen F. Altschul, John C. Wootton, Elena Zaslavsky, Yi-Kuo Yu

**Affiliations:** 1 National Center for Biotechnology Information, National Library of Medicine, National Institutes of Health, Bethesda, Maryland, United States of America; 2 Center for Translational Systems Biology and Department of Neurology, Mount Sinai School of Medicine, New York, New York, United States of America; Cornell University, United States of America

## Abstract

Most pairwise and multiple sequence alignment programs seek alignments with optimal scores. Central to defining such scores is selecting a set of substitution scores for aligned amino acids or nucleotides. For local pairwise alignment, substitution scores are implicitly of log-odds form. We now extend the log-odds formalism to multiple alignments, using Bayesian methods to construct “BILD” (“Bayesian Integral Log-odds”) substitution scores from prior distributions describing columns of related letters. This approach has been used previously only to define scores for aligning individual sequences to sequence profiles, but it has much broader applicability. We describe how to calculate BILD scores efficiently, and illustrate their uses in Gibbs sampling optimization procedures, gapped alignment, and the construction of hidden Markov model profiles. BILD scores enable automated selection of optimal motif and domain model widths, and can inform the decision of whether to include a sequence in a multiple alignment, and the selection of insertion and deletion locations. Other applications include the classification of related sequences into subfamilies, and the definition of profile-profile alignment scores. Although a fully realized multiple alignment program must rely upon more than substitution scores, many existing multiple alignment programs can be modified to employ BILD scores. We illustrate how simple BILD score based strategies can enhance the recognition of DNA binding domains, including the Api-AP2 domain in *Toxoplasma gondii* and *Plasmodium falciparum*.

## Author Summary

Multiple sequence alignment is a fundamental tool of biological research, widely used to identify important regions of DNA or protein molecules, to infer their biological functions, to reconstruct ancestries, and in numerous other applications. The effectiveness and accuracy of sequence comparison programs depends crucially upon the quality of the scoring systems they use to measure sequence similarity. To compare pairs of DNA or protein sequences, the best strategy for constructing similarity measures has long been understood, but there has been a lack of consensus about how to measure similarity among multiple (i.e. more than two) sequences. In this paper, we describe a natural generalization to multiple alignment of the accepted measure of pairwise similarity. A large variety of methods that are used to compare and analyze DNA or protein molecules, or to model protein domain families, could be rendered more sensitive and precise by adopting this similarity measure. We illustrate how our measure can enhance the recognition of important DNA binding domains.

## Introduction

Protein and DNA sequence alignment is a fundamental tool of computational molecular biology. It is used for functional prediction, genome annotation, the discovery of functional elements and motifs, homology-based structure prediction and modeling, phylogenetic reconstruction, and in numerous other applications. The effectiveness of alignment programs depends crucially upon the scoring systems they employ to evaluate possible alignments. For pairwise alignments, scores typically are defined as the sum of “substitution scores” for aligning pairs of letters (amino acids or nucleotides), and “gap scores” for aligning letters in one sequence with null characters between letters in the other. Substitution and gap scores may be generalized to multiple alignments, i.e. those involving three or more sequences.

Most useful local pairwise alignment algorithms allow gaps and explicitly assign them scores [1]–[4]. However, many local multiple alignment algorithms do not allow gaps, or allow them only implicitly as spacers between distinct ungapped alignment blocks. Indeed the alignments recorded in some protein family databases are explicitly constructed with ungapped alignment blocks separated by variable length spacers [5], and it has been argued that this formalism corresponds well to the observed relationships imposed by protein structure [6]. Short ungapped blocks are also used in the DNA context, to represent, for example, transcription factor binding sites.

Many pairwise substitution scores have been developed for protein [7]–[20] and DNA [21], [22] sequence comparison, and a statistical theory for substitution scores has been developed for local alignments without gaps [23], [24]. It is not trivial to generalize pairwise scoring systems to multiple alignments, and the following four principal approaches have been proposed to this long-standing problem: A) *Tree scores*. An evolutionary tree can be defined relating the sequences in question, with each sequence residing at one leaf of the tree. By reconstructing letters at the internal nodes of the tree, the score for an aligned column of letters is defined as the sum of pairwise substitution scores for all edges of the tree [25], [26]. B) *Star scores*. As a special case of tree-scores, a single “consensus” letter can be defined for an alignment column. The column score is defined as the sum of pairwise scores for the consensus letter to each letter in the column. The tree in question reduces to a star, with the consensus at the central node. C) *Sum-of-the-Pairs or SP scores*. A column score can be constructed as the sum of substitution scores for all pairs of letters in the column [27], [28]. D) *Entropy scores*. Scores can be based on the entropy of the letter frequencies observed in a column [29]; these scores have become particularly popular for DNA alignments. All these approaches are open to refinement, for example by weighting the pairwise scores of the sequences involved.

All reasonable substitution scores for pairwise local alignment are implicitly log-odds scores [23], [30], which compare the probabilities of aligning two letters under models of relatedness and non-relatedness, and the most popular are explicitly so constructed [7], [8], [14]. We argue that multiple alignment column scores should be similarly constructed, based upon explicit target frequency predictions for columns from accurate alignments of related sequences. For this purpose, we propose, the method with the strongest theoretical foundation relies upon the specification of a Bayesian prior, over the space of multinomial distributions for describing alignment columns representing true biological relationships [31], [32]. We call column scores based on such a formalism “Bayesian Integral Log-odds” or BILD scores. Although these scores are implicit in earlier work, their full generality and utility has not been recognized. They may be calculated efficiently, and may be generalized to allow for the differential weighting of sequences in a multiple alignment. We also consider an alternative approach that allows log-odds column scores to be derived from any pairwise substitution matrix.

Given their form, multiple alignment log-odds scores can be used directly to define the proper extent of multiple alignment blocks, and to derive natural scores for profile-profile comparison. We show that they also arise from the perspective of the Minimum Description Length Principle [33], which allows them to be combined naturally with other information theoretic measures. Other direct applications are specifying when a sequence should be included in a multiple alignment at all, and when an alignment of many related sequences is better split into several alignments each involving fewer sequences.

Efficient methods for calculating BILD scores allow them to be incorporated into Gibbs sampling algorithms for ungapped local multiple alignment. Most practical protein applications, however, require provisions for gaps. We describe two methods for extending an ungapped local multiple alignment produced by the Gibbs sampling strategy to a gapped alignment, the first using asymmetric affine gap costs, and the second hidden Markov models. In the latter, column BILD scores inform the construction of position-specific gap costs, and yield gapped alignments in greater conformity with considerations of protein structure. We illustrate the applications of the programs by using them to uncover previously undescribed Api-AP2 domains of *Toxoplasma gondii* and *Plasmodium falciparum*.

Multiple sequence alignment comprises a diverse set of problems and approaches. Many sophisticated statistical inference techniques have been applied to the multiple alignment problem and to the related problem of phylogenetic reconstruction, e.g. [34]–[37]. It is not our purpose here to develop a new multiple alignment program. Rather, we seek only to argue that the “substitution scores” for multiple alignment columns which lie at the core of most multiple alignment methods can in many cases be improved. Although many statistical alignment methods are Bayesian-based, the BILD scores directly implied by Bayesian reasoning have been heretofore unrecognized.

## Methods

### Multiple Alignment Log-Odd Scores

Log-odds pairwise substitution scores can be written 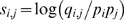. Here, 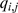 is the frequency with which residues 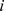 and 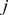 correspond in accurate alignments of related sequences, and 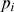 is the background probability with which residue 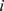 occurs. The base of the logarithm is arbitrary, and merely defines a scale for the scoring system. We henceforth assume that unless the natural logarithm is specified, all logarithms are base 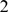, and the resulting scores are therefore in the units of bits [30]. Note that no target frequencies 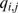 are uniquely optimal for pairwise sequence alignment, because different 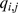 are appropriate for comparing sequences diverged by different amounts of evolution [7], [8], [13], [30]. This perception gives rise to families of substitution matrices, such as the PAM [7], [8] and BLOSUM [14] series for protein comparison.

To generalize log-odds scores to multiple alignments, we first develop some notation. We consider the alphabet 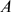 from which the letters in our sequences are drawn to consist of 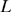 elements, which for convenience we represent by the numbers 1 through 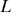. An ungapped column from a multiple alignment of 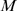 sequences is a vector 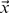, each of whose components 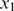 through 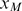 takes on a value in 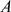. In essence, the log-odds approach compares two theories, one in which all the letters aligned are related or homologous, and the other in which none are. Each theory implies a probability for observing any given set of data. For the alignment column 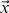, we define 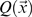 as the probability of observing the data under the assumption of relatedness, and 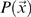 under the assumption of non-relatedness. Then the log-odds score for this column is defined as
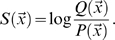
(1)Assuming background probabilities 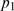 through 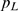 for the various letters, 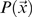 is given simply by
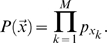
(2)We will consider one primary strategy for deriving 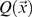. As with pairwise scores, all sets of multiple alignment column scores with negative expected value are implicitly log-odds scores [23], [30]. However, unless their values for 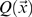 are explicitly constructed in a sensible way, log-odds scores are unlikely to perform well in the applications suggested below.

For alignments of more than two sequences, there are of course other possibilities than for all or none of the sequences to be related. However, as we will describe below, scores of the form of equation (1) can be applied to the comparison of sequences where only a subset are related, by adding indicator variables to include or exclude sequences.

Log-odds scores 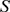 for alignment columns immediately suggest substitution scores 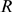 for aligning two different columns of letters. Specifically, letting 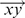 be the concatenation of the vectors 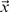 and 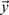, define
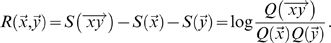
(3)These column-column alignment scores may be used consistently in progressive alignment algorithms, which proceed by aligning the most closely related sequences first [38], [39], although as will be discussed below problems may arise in the definition of gap scores. They may also be used for profile-profile alignment, a topic of considerable recent interest [40]–[48].

### BILD Scores

For multiple alignments, perhaps the best approach to defining and calculating 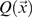 is a Bayesian one [31], [32]. (An alternative approach, based on pairwise scoring matrices, is described in Text S1.) Assume that the letters in a specific column from an accurate alignment of related sequences are generated independently, but with probabilities 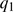 through 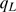 that in general differ from the background probabilities. Assume further that it is possible to assign a prior probability distribution 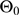 to the multinomial distributions 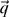 associated with columns of related letters. This prior 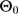 can be derived from a detailed study of related protein or DNA sequences.

Although the data 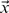 associated with a specific column generally have no temporal or other privileged order, assume for convenience that they are observed sequentially, in the order 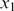 to 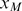. Then we may apply Bayes' theorem to transform the prior distribution 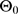 to a posterior 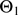, after the observation of 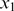. More generally, each subsequent observation 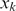 can be seen to transform the prior 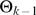 into a posterior distribution 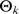. We may then use the chain rule to write
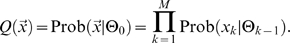
(4)The individual terms in this product may be calculated by integrating over all possible multinomial distributions 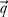:
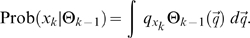
(5)Finally, combining equations (1), (2) and (4) yields
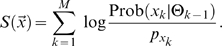
(6)


We call scores defined in this way Bayesian Integral Log-odds or BILD scores. They can be understood simply as the sum of log-odds scores for the individual letters observed in a column, with the “target frequency” for each letter 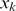 calculated based upon the prior distribution 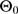, and the “previously observed” letters 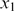 through 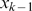. Even though, by this formula, the log-odds score for a letter varies with its position in the column, the total column score is nevertheless invariant under permutation of the column's letters.

BILD scores have some conceptual connections to star- and entropy-based multiple alignment scoring systems. The simplest generalization of star scores imposes a prior probability distribution on the consensus letter, but still assumes a probabilistic pairwise substitution model. As we describe in Text S1, this yields a class of log-odds scores we call MELD scores. BILD scores arise, in contrast, by thinking of the “consensus” not as an ancestral letter, but rather as a generative probabilistic model, and by integrating over a prior distribution placed on this model.

Given observed and background letter distributions 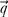 and 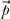, entropy scores have been defined variously, and conceptually distinctly, as: i) 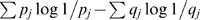, the entropy difference between 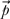 and 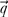; ii) 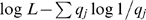, the entropy difference between a uniform distribution on 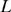 letters and 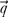; and iii) 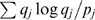, the relative entropy of 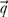 and 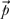. Definitions i) and ii) differ only by a constant. One may refine any of these definitions by taking 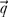 to be a posterior letter distribution, derived from a prior and a set of observations. Both BILD and entropy-based scores can be viewed as the sum of scores derived from the probabilities for individual observations. The central distinction is that BILD scores estimate the probability for a given such observation using only “earlier” ones, whereas entropy scores estimate this probability using the complete collection of observations.

### Dirichlet Distributions

Although the definition of BILD scores is valid for any prior distribution 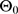 one wishes to specify, it is in general impractical to calculate the 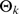, or the integral in equation (5), except when 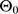 takes the form of a Dirichlet distribution [49], or a mixture of a finite number of Dirichlet distributions [31], [32]. In this case, as described below, all the 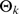 are also Dirichlet distributions, or Dirichlet mixtures, and 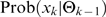 is easily calculated. Therefore, for mathematical as opposed to biological reasons, we always assume that BILD scores are defined using a Dirichlet or Dirichlet mixture prior. The family of Dirichlet mixtures, however, is rich enough that it can capture well much relevant prior knowledge concerning relationships among the various amino acids or nucleotides.

We review here the essentials of Dirichlet distributions. A multinomial distribution on 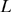 letters is specified by an 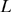-dimensional vector 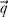, within the simplex defined by 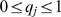, and 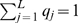. The requirement that the 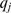 sum to 1 renders the space of multinomials 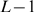 dimensional. A Dirichlet distribution, defined over this space, is parametrized by an 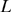-dimensional vector 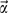 with all 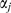 positive. We shall sometimes refer to such a distribution by its parameters 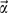, and we define 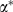 as the sum of the 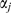. The Dirichlet distribution 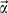 is given by the probability density function
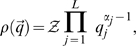
(7)where the normalizing scalar 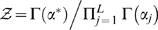 ensures that integrating 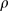 over its domain yields 1. Here 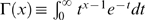, is the Gamma function, and 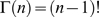 for positive integral 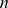. The uniform density is a special case that arises when all the 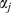 are 1.

Dirichlet distributions have two convenient properties. First, the expected frequency of letter 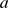 implied by 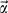 is 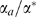. Second, the posterior distribution yielded by Bayes' theorem, after the observation of the letter 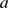, is a Dirichlet distribution 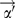 with 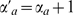, but with all other parameters equal to those of 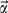.

To illustrate how to calculate BILD scores using these properties, consider the case of DNA comparison (with the numbers 1 through 4 identified respectively with the nucleotides A, C, G and T), with uniform background probabilities 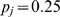, and a Dirichlet prior 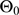 given by the parameter vector (1,1,1,1). By equation (4), the target frequency 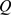 associated with the alignment column “AATC” is given by 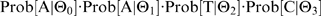 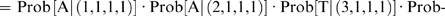 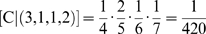. Thus the score for the column is 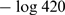 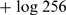 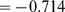 bits. In contrast, for the column “AAAC”, 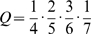, and the score for this column is 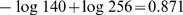 bits.

The essence of a Dirichlet distribution is perhaps best understood through the alternative parametrization (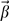; 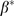), where 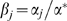, and 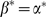. Because the 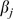 must sum to 1, there are still only 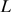 independent parameters. The vector 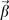 describes the center of mass of the distribution, while 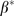 indicates how concentrated the distribution is about this point. Large values of 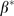 correspond to distributions with most of their mass near 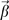, whereas values of 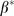 near 0 correspond to distributions with most of their mass near the boundaries of the simplex. It is frequently sensible, although not necessary, to choose a prior 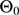 whose 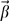 is identical to the background frequencies 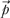. In this case, 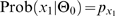, and the first summand in equation (6) is always 0. In other words, no letter in a column, considered in isolation, carries any information as to whether the column represents a true biological relationship.

### Dirichlet Mixtures

Single Dirichlet distributions frequently are adequate for capturing prior knowledge concerning “true” alignment columns of related DNA sequences, but this is not the case for proteins. Most simply, distinct regions of multinomial space, representing different collections of amino acids, should have high prior probabilities. In order to address the deficiency of single Dirichlet distributions, Brown 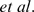 [31] proposed the use of Dirichlet mixture priors. A Dirichlet mixture is simply the weighted sum of 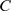 distinct Dirichlet distributions. It is specified by 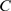 positive “mixture parameters” 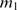 through 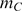 that sum to 1, and a set of 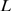 standard Dirichlet parameters, 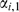 through 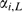, for each of the 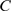 component Dirichlet distributions. (It will be useful later to define 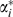 as 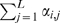.) In all, because of the restriction on the sum of the 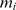, a Dirichlet mixture has 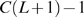 independent parameters. The Dirichlet components of a mixture generally are thought of as describing various types of positions (e.g. hydrophobic, charged, aromatic) typically found in proteins.

Bayes' theorem implies that, given a 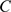-component Dirichlet mixture as a prior, the posterior distribution after the observation of a single letter is also a 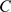-component Dirichlet mixture [31], [32]. Brown 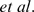 [31] proposed Dirichlet mixture priors in the context of deriving “substitution” scores for aligning amino acids to columns from a multiple protein sequence alignment. This restricted context can be understood as comprehending a single summand from equation (6). BILD scores extend Brown 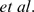
's sequence-profile alignment scores to comprehensive scores for multiple alignment columns.

Generalizing the development above, we describe here how to calculate the probability of a particular observation 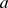 given a Dirichlet mixture prior 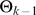, and how to calculate the posterior 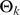 resulting from this observation. First, given a Dirichlet mixture, with parameters 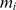 and 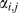, the probability of observing letter 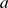 is given simply by
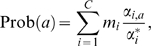
(8)which follows directly from the definition of Dirichlet mixtures, and the result for single Dirichlet distributions. Second, given the observation of letter 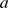, and a Dirichlet mixture prior parametrized as above, the parameters 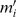 and 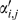 of the posterior distribution may be calculated as follows:
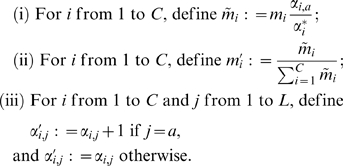
(9)In short, first multiply the mixture parameters 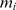 by the Bayesian factors 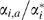 and normalize, and then add 1 to each 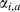. Mathematics establishing the validity of this procedure appears in [32]. Their development is more complex than we require here, because we modify the Dirichlet mixture parameters only one observation at a time. We note that given the 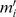 and 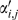, it is simple to invert procedure (9) to determine the 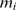 and 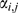. This is useful for applications such as the Gibbs sampling algorithm discussed below.

Many multiple alignment problems involve subsets of sequences that are much more closely related to one another than to the other sequences being considered, and this may yield suboptimal results, because a large number of closely related sequences can “outvote” a few more divergent sequences. One remedy has been to assign each sequence a numerical weight, with closely related sequences down-weighted [50]–[61]. Also, subsumed in such weights may be the recognition that the total number of effective observations represented by an alignment column may be smaller than the number of sequences it comprehends [4], [62], [63]. Thus, for certain applications it may be desirable to generalize BILD scores to weighted sequences. To do so, we need to define the concept of the probability of a “fractional observation” of a letter, and describe as well how a posterior distribution is calculated after such a fractional observation. Arguments supporting how this may be done can be extracted from the mathematical development in [32]. Both equation (8) and the first step of procedure (9) involve multiplication by the factors 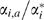. For the fraction 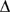 of an observation of letter 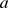, these factors must be replaced by the alternative factors
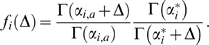
(10)Also, in the last step of procedure (9), the quantity 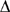 rather than 1 must be added to each 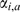. The factors 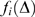 are identical to the original factors when 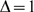, and all 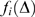 approach 1 as 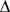 approaches 0, as some reflection shows they must.

Finally, note that equation (10) may be applied to 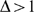 as well as 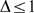, and may be useful even when all observations are unitary. Thus, by aggregating observations, the BILD score for a column containing 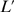 unique letters may be calculated with 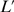 summands, rather than the 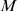 summands of equation (6). For a single Dirichlet prior, 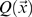 reduces to the simple formula
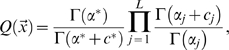
(11)where 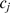 is the count of letter 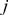, and 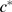 is the total count of all residues. Only the numerator inside the product varies from column to column within an alignment, yielding further efficiency for calculation.

### The Choice of Priors

Only the research team that first proposed Dirichlet mixtures for protein sequence comparison has derived, from analyses of large protein alignment collections, sets of Dirichlet mixture prior parameters [31], [32]. Twelve such sets, involving various numbers of Dirichlet components, can currently be found at http://compbio.soe.ucsc.edu/dirichlets/index.html. We list five of these in Table 1, which we refer to as 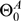 through 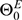.

**Table 1:** Dirichlet mixture priors for protein sequence comparison.

Name of prior	Name on UCSC website	Number of components	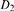 (bits)	Equivalent PAM matrix	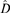 (bits)	Equivalent PAM matrix
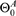	uprior	9	1.44	80	2.85	20
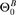	byst	9	0.91	130	2.34	35
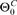	recode3	20	0.61	175	1.88	55
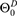	recode4	20	0.37	245	1.63	70
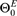	fournier	20	0.18	360	0.92	125

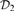
 is the relative entropy [30] of the pairwise substitution matrix implied by the Dirichlet mixture prior. 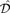 is the mean relative entropy of the multinomial distribution (Text S2).

Proteins diverged by different degrees of evolutionary change are best studied using pairwise substitution matrices with different relative entropies [30], and the analogous claim should hold for Dirichlet mixture priors. A Dirichlet mixture prior implies a background amino acid frequency distribution 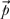, as well as a symmetric pairwise substitution matrix, by means of the formula 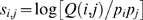. The relative entropies 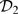 of the substitution matrices implicit in the priors 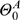 through 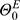 range from 1.44 bits, roughly equivalent to that of the PAM-80 matrix [7], [8], which is appropriate for fairly close evolutionary relationships, to 0.18 bits, roughly equivalent to that of the PAM-360 matrix, which is appropriate only for extremely distant relationships (Table 1).

As well as 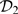, one may calculate the mean relative entropy 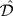 of the multinomial distributions 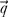 described by a Dirichlet mixture prior to the background frequencies 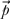 (see Text S2). For 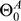 to 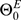, 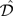 ranges from 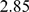 to 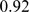 bits (Table 1). That 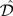 has a much greater value than 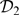 indicates that on average much more information is available per position from an accurate multiple alignment of many related sequences than from a single sequence. We note that, in lieu of using different priors, the effective relative entropy of a particular Dirichlet mixture may be tuned by scaling the weights of the sequences to which it is applied [43].

Standard pairwise substitution matrices are constructed from sets of proteins with certain background amino acid frequencies 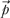, and are non-optimal for the comparison of proteins with compositions that differ greatly from 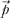 [64]. Similarly, a Dirichlet mixture prior has an implicit background amino acid composition 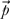, and should not be optimal when applied to proteins with compositions that differ greatly from 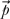. It is possible to adjust standard matrices for use with non-standard compositions [64], [65], and we will discuss elsewhere an analogous strategy that can be applied to adjust Dirichlet mixture priors.

Single Dirichlet priors may be appropriate for DNA sequence comparison. The uniform density, arising when all 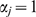 (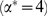), has frequently been advocated in the absence of prior knowledge, and “Jeffreys' prior” [66], which is uninformative in a deeper sense, corresponds to all 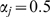 (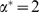) [33]. When specific prior knowledge concerning an application domain is available, however, there is generally not a strong argument for using uninformative priors. For related DNA sequences, the columns of accurate alignments are sometimes dominated by one or two nucleotides, suggesting that all 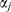 should be smaller than 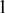. Furthermore, it usually makes sense for the 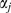 to be proportional to the background frequencies 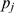. If this is stipulated, the specification of a Dirichlet prior reduces to the specification of 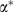. Assuming a uniform nucleotide composition, the values of 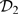 and 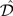 implied by 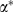 from 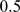 to 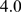 are given in Table 2. An empirical study of transcription factor binding sites [67] concludes that, at least for the analysis of such sites, 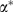 should be 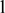 or lower.

**Table 2:** Relative entropies for DNA sequence comparison.

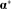	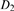 (bits)	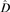 (bits)
0.5	0.792	1.387
1.0	0.451	1.062
1.5	0.294	0.860
2.0	0.208	0.721
2.5	0.155	0.621
3.0	0.120	0.545
3.5	0.096	0.485
4.0	0.078	0.437

See footnote to Table 1.

### Local Alignment Width and Local Multiple Alignment

A direct application of multiple alignment log-odds scores is to determining local alignment width. As formulated by Smith and Waterman [1], an optimal local alignment is one that maximizes an alignment score but is of arbitrary width. Such scores should fall on the log side of the “log-linear phase transition” [68], which implies that for ungapped local alignments, substitution scores must be of log-odds form [23], [30].

Equation (1) explicitly generalizes pairwise log-odds scores to the multiple alignment case. They are positive for some alignment columns, negative for others, and must have negative expected value. Therefore it is appropriate to define an optimal ungapped multiple alignment as one with maximal aggregate log-odds score. This immediately allows one to define the proper width or extent of an ungapped multiple DNA or protein alignment, without resorting to the *ad hoc* principles frequently required for other scoring systems [69]. Although the Smith-Waterman algorithm can be applied to optimize log-odds-scored local multiple alignments, it is too slow for most purposes. Nevertheless, once relative offsets have been fixed for a set of sequences, it is trivial to determine an optimal ungapped local multiple alignment along the single implied diagonal.

The ungapped local multiple alignment problem may be formulated as seeking segments of common width 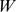 within multiple DNA or protein sequences that, when aligned, optimize a defined objective function. We take this function here to be the aggregate log-odds score for the aligned columns. One way to approach this optimization is by means of a Gibbs sampling strategy, as described by Lawrence 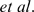 [69]. Log-odds scores can be used to adjust 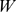 dynamically, by applying the Smith-Waterman algorithm to the diagonal implied by a provisional alignment, without the need for an arbitrary parameter or an *ad hoc* optimization. They may also be used to determine dynamically whether or not a sequence should participate in the multiple alignment at all, for which purpose it is useful first to consider log-odds scores from the perspective of the Minimum Description Length Principle.

### Log-Odds Scores and the Minimum Description Length Principle

The Minimum Description Length (MDL) Principle provides a criterion for choosing among alternative theories for describing a set of data [33], [49]. To simplify greatly, it suggests that given a set of alternative theories 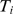 to describe a set of data 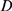, that theory should be chosen which minimizes 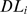, defined as the sum of 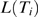, the description length of the theory, and 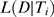, the description length of the data given the theory. By convention, description lengths are measured in bits.

From information theory [70], the information associated with an event of probability 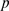 is 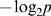 bits. Focusing on actual encoding schemes for probabilistic events can unduly complicate MDL analyses. Accordingly, we here follow the approach of section 3.2.2 of [33], in which description lengths are allowed to be non-integral, and are identified with negative log probabilities. Thus, if the data can be described probabilistically, 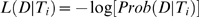. The length of the theory 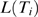 is defined as the number of bits needed to specify the free parameters of 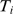, i.e. those that are fitted to the data [33].

For local multiple alignment, the theory 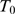 that the input sequences are unrelated has only the background probabilities 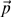 as parameters, whose description length we will call 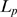. The data 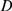 is comprised of 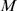 sequences, with lengths 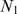 through 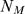, and consisting of the letters 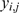. Then 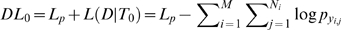. The theory 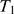 states that segments of width 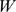 beginning at positions 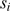 within the various sequences are related, and that the probability of the data 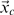 within each column 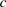 of the implied alignment is 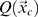; the probability of the rest of the data may be described with the background frequencies 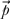. The free parameters are 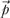, the vector of starting positions 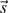, and 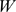. Each 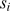 may take on one of 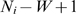 values, so its description length is approximately 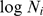, if 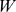 is not too large compared to 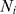. Thus, we have 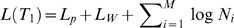, where 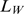 is the description length of 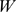. (If all feasible widths are taken to be equally likely, 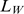 is just 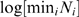. Other encodings have 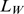 grow slowly with 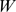 [33], [49].) It is apparent that 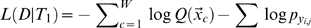, where the latter sum is taken only over those letters not participating in the local multiple alignment. Everything simplifies when we consider the difference in the total description lengths of the two theories:
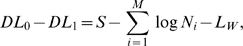
(12)where 
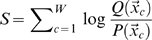
 is simply the log-odds score for the implied alignment. In other words, 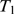 is preferred whenever 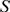 exceeds 
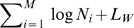
. As described in Text S3, this prescription is related to the statistical theory for ungapped local alignments [23].

To allow one or more sequences to be excluded from the multiple alignment, we consider not 2, but 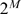 theories, distinguished by 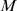 binary indices 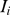, which take on the value 1 to indicate that sequence 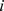 participates in the alignment, and 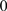 otherwise. These theories need not be *a priori* equally likely; if necessary, for 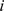 from 1 to 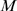 we can specify prior probabilities 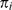 that sequence 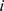 contains a segment related to segments in the other sequences. Let us consider the difference in the description lengths of two theories, 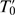 and 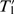, that differ only in their index 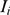. Theory 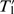 incurs the cost 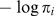 for the prior probability that 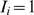, and also requires describing the location of the related segment, which costs 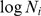 bits. In contrast, theory 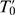 incurs only the cost 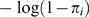, so 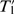 costs 
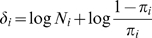
 more bits to describe than 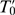. Thus, for 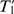 to be preferred, the log-odds score of the multiple alignment must increase by at least 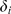 when the segment from the 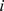
th sequence is added. If 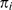 is close to 1, 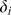 can be negative, and is 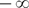 if 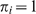. In short, the greater the prior probability that a given sequence contains a relevant segment, the lower the score of such a segment need be for inclusion in the alignment.

The change in the log-odds score with the addition of a segment from the 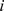
th sequence depends upon which other sequences, and which of their segments, participate in the alignment. Consequently, the values of the indicator variables 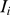 must be part of the larger optimization, and their selection can be readily incorporated into a Gibbs sampling algorithm. The MDL Principle can also be extended to the case where a single sequence may contain more than one copy of a pattern, and, as previously described [62], [71], [72] and discussed in Text S4, to the clustering of multiple alignments into subfamilies.

### Gap Scores

Although our central concern is to define a new type of multiple alignment substitution score, many important applications require the construction of gapped multiple alignments, and these generally entail scores for insertions and deletions. Multiple alignment gap scores should be defined in a manner consistent with the substitution scores used [73], so we will consider what type gap scores might fruitfully be paired with BILD scores.

Just as the log-odds perspective places pairwise substitution scores in a probabilistic framework [7], [8], [23], [30], so pairwise gap scores can be viewed as specifying probabilities for insertions and deletions within biologically accurate alignments [74]–[82]. For pairwise alignments, “affine” gap scores, of the form 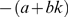 for a gap of length 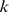 [83]–[85], are those most commonly used [3], [4], although more complex gap scores have frequently been proposed [86]–[89]. When there is an essential asymmetry between the sequences being aligned, differing scores may be assigned to gaps within the two sequences. Furthermore, when substitution and gap scores are properly integrated and both expressed in the units of bits, the two parameters of affine gap scores can be understood to specify jointly the average frequencies and lengths of gaps in the alignments sought [82]. If gaps are to be introduced into the BILD score formalism, an immediate problem is which, if any, letters from individual sequences should be understood as insertions with respect to the “canonical” pattern. In other words, it appears a canonical width for the multiple alignment must somehow be chosen, with respect to which gaps arising in the alignment of individual sequences can be assessed.

### Profile-Sequence Alignment

For simplicity, suppose we have a “canonical” multiple alignment 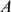, i.e. one with a specified number of columns, to which we wish to align a single sequence 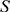, to produce a new multiple alignment 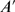. It is reasonable to define the alignment score of 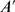 as the pre-existing alignment score for 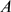 plus the incremental pairwise score for aligning 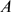 and 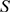. This pairwise alignment involves substitutions (letters from 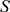 aligned to columns from 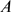), insertions (runs of letters from 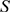 that are not aligned to any columns from 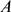), and deletions (runs of columns from 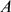 that are not aligned to any letters from 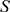). BILD scores for the columns of 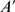 arise naturally when one defines the substitution scores for aligning 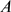 to 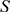 as incremental BILD scores. It remains then only to define gap scores for insertions and deletions in the alignment of 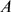 and 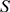.

There is an essential asymmetry in gap scores for aligning 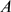 to 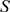, relevant in many biological applications. For proteins, the columns of 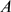 represent canonical positions, present in most sequences of a protein family, and it should accordingly be very costly to delete any of these columns. In contrast, individual proteins often contain long loops not present in the great majority of related sequences [90], [91], so even long insertions should not be very costly. Uniform but asymmetric affine insertion and deletion scores can capture this simple idea, and we have implemented them in one program described in the Results section below. These scores can be derived from the average frequencies and lengths [82] of insertions and deletions with respect to canonical protein family multiple alignments.

Just as incremental BILD substitution scores change as more sequences are added to a multiple alignment, so it is possible to let insertion and deletion scores change as well, and vary by position. In the context of Hidden Markov Models [76]–[81], many methods for doing this have been described. Below, we implement one simple procedure that depends only upon the BILD scores of multiple alignment columns, and not upon the relatively sparse gaps observed in any particular alignment.

### Progressive Multiple Alignment and Profile-Profile Alignment

Formula (3) permits BILD substitution scores to be used for progressive multiple alignment. However, as described above, gaps scores pose a particular problem, because to define insertions and deletions one needs to construct a canonical alignment, and this is difficult for a small number of sequences. For example, when just two proteins are aligned, it is quite possible that gaps in both sequences would ultimately be seen as insertions with respect to a model describing the whole protein family, but there is no obvious way to determine this in advance. (The problem does not arise when substitution and gap scores are defined using the sum-of-pairs or SP formalism [27], [28], for which no canonical alignment is necessary [73].) Accordingly, the approach we take below is eschew gaps at first, and thereby construct a canonical multiple alignment whose columns represent positions present in the majority of sequences. Only then do we realign individual sequences to this model, allowing gaps.

There has been considerable recent interest in aligning profiles that describe different protein families [40]–[48]. If BILD substitution scores, defined by equation (3), are to be used for this purpose, it would seem that we face the same problem for gaps that we do for progressive multiple alignment. Specifically, an insertion with respect to one profile is seen as a deletion with respect to the other, so how may one determine which, if either, perspective to adopt in a model describing both? However, so long as this goal is only to compare pairs of profiles, and not to proceed further, this problem may be elided. It is consistent to define pairwise gap costs for the alignment of two profiles, just as one would for the alignment of two sequences, without reference to a canonical alignment, and the substitution scores of equation (3) can be used sensibly with such gap costs. The gap costs chosen may depend upon the profiles being aligned, and may therefore be asymmetric and position specific. We leave for elsewhere the comparative evaluation of profile-profile alignment using substitution scores defined by equation (3), and those defined in other ways [40]–[48].

## Results

Substitution scores for multiple alignment columns form only one element of successful multiple alignment programs. Depending upon their specific purposes, such programs may also employ gap scores, sequence weights, heuristic optimization algorithms, low-complexity filters, discontiguous patterns, provisions for no or multiple copies of a pattern within a sequence, the search for multiple distinct patterns, statistical assessments, etc. It is not our purpose here to develop a fully realized program to outperform existing state-of-the-art programs that involve multiple alignment. Rather, we seek only to argue that the use of explicitly constructed log-odds substitution scores can in many cases add values to these methods.

The programs we consider below have been constructed for evaluation purposes, to isolate the contribution of log-odds scores as much as possible. These programs are parsimonious in their complexity and use of free parameters, and employ various ideas that have appeared frequently elsewhere, and for which no novelty is claimed.

### A. Ungapped Multiple Local Alignment Using Gibbs Sampling

BILD scores find perhaps their purest application in the ungapped local alignment problem described above, so it is worth studying them in this restricted context. The Gibbs sampling approach to finding optimal local multiple alignments was introduced by Lawrence et al. [69], and this algorithm can easily be modified to employ BILD scores. Potential advantages are improved sensitivity and the automatic definition of domain boundaries. Evaluation ideally requires a set of proteins with ungapped domains whose correct alignment is structurally validated, but such sets are unfortunately very rare. Nevertheless, the collection of ungapped helix-turn-helix (HTH) domains in [69] provides a limited test set for analyzing BILD scores in the absence of gaps. As we describe in Text S5, with Tables S1 and S2, BILD scores achieve success on two fronts. First, they have greater average sensitivity than the entropy-based scores proposed by Lawrence et al. [69], in yielding accurate alignment from fewer sequences; second, they recognize with good precision the extent of the structurally-defined domains, and therefore do not require a prior specification of alignment width.

### B. Extension to Gapped Local Alignment

Local multiple alignment programs generally must allow for gaps, either implicitly or explicitly. However, even for aligning gapped domains, the search for ungapped local alignments can be a fruitful first step. BILD scores can play an important role at this stage in defining the common core of a protein family, and can be adapted in subsequent stages to score gapped multiple alignments. As a proof of principle, we here develop a relatively simple algorithm, Program 1, that uses BILD scores as part of a gapped multiple alignment strategy. We describe this program's architecture and motivation below, and use a standard artificial test set to evaluate its ability to recognize the boundaries of local motifs, and to properly construct gapped local alignments. We then describe in section C how Program 1 may be refined through the consideration of features of protein structure, and illustrate the application of our methods to the delineation of a protein domain family.

#### Program 1 architecture

Input: A set of putatively related protein sequences potentially containing zero, one, or multiple instances of a common pattern. The sequences are in a standard unaligned format such as fasta.

Goal: To find a gapped local multiple alignment that optimizes an objective function defined as the sum of column BILD scores, minus gap costs, minus costs for describing the start locations of patterns. The user may specify whether a single or multiple instances of the pattern in each sequence should be sought, as well as whether the pattern may be absent in some sequences.

Heuristic algorithm:

- Execute the Gibbs sampling strategy (Text S5) to determine a preliminary pattern width, and a preliminary ungapped local alignment, allowing at most one instance of the pattern per sequence.
- For each input sequence 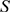, remove any and all segments of 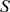 from the multiple alignment, and construct a BILD-score based position-specific score matrix (PSSM) 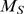 from the remaining alignment. Allowing gaps with affine gap costs [83], [85], optimally align the whole of 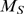 to a segment from 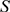, using for this purpose a generalization of the semi-global alignment algorithm of Erickson and Sellers [92]. Consider all sequences 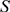, whether or not they were identified as containing a pattern in the initial Gibbs sampling stage, or in subsequent gapped alignment iterations. Asymmetric gap costs for insertions and deletions may be specified. (Note that, as described in the hidden Markov model (HMM) literature [76]–[82], for bit scores to retain their meaning, a small penalty, equivalent to the log probability of *not* initiating a gap, must be assessed whenever a letter is aligned to a motif column. For our purposes, this penalty is best viewed as an additional “gap score”, although it may be coded as a modification to the substitution scores. Also, when a letter is not aligned to a motif column, the number of observations and aggregate BILD score for that column do not change.) Retain the alignment if the score exceeds a calculated threshold. Multiple non-overlapping segments within 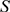 that align to 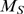 can be found using a greedy approach.
- Collect all the aligned segments from step b) into a new, gapped multiple alignment, and return to step b). Iterate until the objective score function stops increasing.
- Adjust the width of the original pattern to optimize the alignment score. Alternatively, this step may be inserted between iterations.

#### Program 1 motivation

The initial search for ungapped segments in the Gibbs sampling step can delineate a common core pattern width, and provisional amino acid frequencies for each column, shared by a set of sequences, even when most segments are at first partially misaligned. For sequences containing repeated or multiple distinct patterns, it may be useful to restrict the width of the pattern sought. The MDL Principle can be used to provisionally exclude some sequences from the alignment at this stage, which may then be included later. Adopting this core pattern generally minimizes the average number of gaps that subsequently need to be introduced when aligning to members of the family. Using Erickson-Sellers semi-global alignment conserves the pattern width 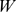, recognizing the importance of complete domains, and thereby both reduces the noise from chance partial similarities and aids the discovery of long insertions. Gapped alignment avoids the imposition of a block structure that may not be universally appropriate. However, columns are not added to the evolving profile to represent insertions, which can be idiosyncratic in length and location. Deletions may be present, but these are generally short and in a small minority of the sequences. Thus, the 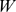 concatenated aligned columns are densely occupied by amino acid data and are highly informative. This compressed type of profile, like the similar representation of Neuwald and Liu [82], generally corresponds well to the core structural elements of a domain. The use of asymmetric gap costs (with greater penalties for deletions) captures the natural asymmetry implied in aligning a sequence to such a core model. Note that elsewhere Gibbs sampling has been extended directly to the construction of gapped alignments [93], [94], whereas Program 1 takes the simpler approach of confining the Gibbs sampling stage to the discovery of a provisional ungapped pattern.

#### Program 1 performance

The evaluation of the performance of a multiple alignment program requires a collection of sequence sets for each of which the correct alignment is known [95], [96]. Multiple alignment programs may focus on the construction of global alignments, or on the discovery of local patterns, and different collections are accordingly appropriate for their evaluation. Among those collections in common use, “ref1” from IRMBase [96], which we will call IRM-1, appears the most appropriate for our gapped local multiple alignment program. IRM-1 contains 60 sets of sequences, with the sequences in each set containing a single, possibly gapped, local motif, embedded within otherwise random sequence. The motifs were generated artificially using the Rose program for simulated evolution [97]. This construction, although not completely realistic, means, however, that the extent and correct alignment of the motifs within the various sequences are precisely known. The 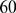 sets are divided into three groups of 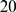, consisting respectively of sets of 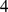, 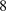 and 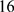 sequences.

First, we evaluated the ability of Program 1 to identify properly the left and right motif boundaries within the 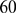 IRM-1 sequence sets. The results, grouped by the number of sequences within the various IRM-1 sets, are shown in Table 3, with positive deviations referring to patterns identified by Program 1 that are too long. Program 1 identifies 80% of the motif boundaries exactly, and 92% to within 1 residue. Furthermore, the accuracy of boundary detection clearly improves as the number of sequences considered increases.

**Table 3:** The recognition of motif boundaries.

Program 1									
	Deviation from true boundary								
IRM-1 subset	<−3	−3	−2	−1	0	1	2	3	>3
4		1	1	5	29	1	2	1	
8				1	31	6	1	1	
16	1		1		36	1			1
Total	1	1	2	6	96	8	3	2	1

DIALIGN-TX									
	Deviation from true boundary								
IRM-1 subset	<−3	−3	−2	−1	0	1	2	3	>3
4	2	2	1	6	23	4	2		
8				6	24	7	2	1	
16	1		3	3	11	10	9	3	
Total	3	2	4	15	58	21	13	4	

Counts were made of the deviations found by Program 1 and DIALIGN-TX of the left and right pattern boundaries (120 total) for the embedded motifs within the 60 IRM-1 sequence sets, divided into the sets involving 4, 8, and 16 sequences [96]. At all 120 boundaries of the reported patterns, both programs align in register at least 50% of the sequences. This consensus allows us to determine to what extent the programs report conserved regions that are too long or too short. Positive deviations in the table refer to patterns identified by the programs that are longer than the actual patterns. To make an equitable comparison of the two programs, several non-default options and procedures were employed, as follows: (1) Asymmetric affine gap costs were inappropriate for Program 1 because the Rose program [97] used in the construction of IRM-1 does not simulate the differential rates with which insertions and deletions occur within real protein motifs. Accordingly, we empirically assigned all gaps of length 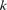 a score of 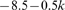 bits, which corresponds [82] to an average frequency of 0.67% for insertions (and similarly for deletions) beginning at each motif position, and an average insertion or deletion length of 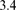. (2) We ran DIALIGN-TX at its least sensitive setting, using the “-l2” option, to avoid the excessive extensions into randomly aligned flanking sequences that degrade the accuracy of motif boundary recognition with the more sensitive default setting. (3) For DIALIGN-TX, we defined the boundary of a conserved motif as the maximum left or right extent to which *all* of the set of sequences aligned in register were reported as conserved. An alternative criterion might be to take a majority vote on the left or right extent of the reported pattern, but this criterion often gave unreasonably long extensions with DIALIGN-TX, and so was not used. For Program 1 run with the 16-sequence input sets, two outliers were found (columns headed 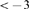 and 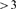). These are cases where roughly half the sequences in the set contained large insertions or deletions, leading Program 1 to misalign a substantial minority of sequences at one of the boundaries.

Most multiple alignment programs do not explicitly identify in their output conserved motifs as distinct from randomly aligned sequence. However, the output of program DIALIGN-TX [98], developed by the same research group that constructed IRM-1, displays the significantly conserved residues within each sequence in upper case letters, although these do not generally fall into completely consistent aligned columns. We have used this feature to compare the performance of DIALIGN-TX at identifying motif boundaries with that of Program 1 (Table 3, with details in the caption). DIALIGN-TX identifies 48% of motif boundaries exactly and 78% to within 1 residue, but its performance appears to degrade as the number of sequences considered increases. In summary, although existing multiple alignment programs such as DIALIGN-TX can do quite a good job at identifying the extent of common motifs embedded within random sequence, the use of BILD scores for this purpose can lead to noticeably improved precision.

The IRM database has been used previously to evaluate the performance of multiple alignment programs by computing how accurately they align the letters that are, by construction, “homologous” [96], [99]. Given a set of sequences 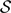 from IRM, and the multiple alignment 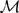 produced by a particular program, the quality score for the program is defined to be the percentage, taken over all pairs of sequences within 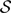, of the homologous pairs of letters, within the annotated IRM-1 motif, that are aligned in 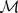 [99]. We used this measure to compare Program 1 to a variety of multiple alignment programs representative of distinct strategies: ClustalW [100]; PCMA [101]; MUSCLE [102], [103]; ProbCons [104]; COBALT [99]; and DIALIGN-TX [98]. For each program, various quality score statistics for IRM-1 are presented in Table 4, along with aggregate program execution time. As can be seen, Program 1 performs better than or comparably to all the other multiple alignment programs, as measured by the various quality score statistics, and also runs substantially faster. Caution should be employed in interpreting Table 4, since Program 1 was explicitly designed for discovering single local patterns within otherwise unrelated sequences, while the other programs were primarily designed to construct global multiple alignments, and some use strategies or parameters that are not well adapted to local multiple alignment.

**Table 4:** Multiple alignment accuracy.

	Quality Score Statistics				
Program	Minimum	Mean	Median	% Perfect	Execution time (sec.)
Program 1	60.7	95.0	99.8	48	18
DIALIGN-TX	37.6	94.2	98.4	38	95
PCMA 2.0	16.7	92.3	98.4	23	376
COBALT	45.6	95.1	98.0	22	303
ProbCons 1.10	16.7	82.8	92.2	27	506
MUSCLE 3.6	0.0	38.0	31.5	3	115
ClustalW 1.83	0.0	8.0	3.9	0	27

Quality score statistics were measured in the 60 sequence sets of the IRM-1 database [96]. “Percent perfect” refers to the proportion of the 60 datasets in which all homologous residues were correctly aligned. All programs were run with default parameters, except that Program 1 and DIALIGN-TX used the parameters detailed in Table 3. Because all programs other than Program 1 produce global multiple alignments as a matter of course, the quality score credits them for aligned residues independently of whether these residues are identified as lying within a conserved region. None of these programs explicitly identifies such regions, although DIALIGN-TX does so implicitly, as described in the caption to Table 3. Accordingly, in order not to artificially handicap Program 1 on this test, we calculated its quality scores by aligning, immediately adjacent to the conserved pattern it identifies within each sequence, and without gaps, all the residues deemed to lie beyond this pattern. In the small fraction of cases where the identified pattern stops short of the boundary of the embedded motif (see Table 3), this can produce a slightly better quality score than the pattern, considered in isolation, would yield. CPU execution times are for programs run on a Dual Pentium 4 Xeon 3.0 GHz CPU Linux computer with 64-bit architecture, and are averaged over three runs.

### C. Protein Structure Considerations

As mentioned above, real protein domains are subject, on average, to much longer insertions than deletions, and this implies the utility of asymmetric affine gap costs for Program 1. The particular costs that are best will depend upon the statistical properties of gaps, and a possible refinement of Program 1 would be to adjust gap costs dynamically. From the analysis of a variety of protein families, we have found empirically that reasonable gap scores to use in conjunction with 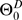 Dirichlet mixture priors are 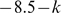 bits for a deletion of 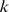 motif positions (corresponding [82] to an initiation frequency per motif position of 0.28%, and a mean length of 2.0), and 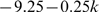 bits for an insertion of length 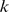 into the motif (corresponding to a frequency of 0.87%, and a mean length of 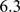).

Protein structure implies more than an asymmetry between the frequency and length statistics of insertions and deletions. Reflecting the evolution of secondary structure elements and loops, certain motif positions are much less likely to be deleted than others and, similarly, insertions are much less likely to occur between certain pairs of motif positions than others. We describe below an extension of Program 1 to an HMM-based Program 2 that relies only upon column BILD scores to calculate position-specific gap score parameters. We then apply Programs 1 and 2 to the detection of Api-AP2 domains.

#### Program 2 motivation and architecture

Protein families or domains are often described by HMMs [76]–[81]. HMMs, in addition to specifying the probabilities for amino acids to occur in various profile positions, may specify distinct probabilities for insertions or deletions to occur in various locations. A more dynamic strategy for model construction than typical for HMMs may be based on the approach described above. As an example of a current strategy, the construction of a Pfam model [105]–[107] starts with a manually-curated gapped multiple alignment of selected members of the protein domain family, the “seed alignment”, from which an HMM profile is built. The seed alignment and HMM are the static canonical entities that define a Pfam family. Then, as a separate procedure, sequence database search programs using this HMM are applied to identify and align additional family members. In contrast, our approach does not entail an initial manual alignment. We start with unaligned sequences, which may include a large proportion of flanking sequence and negative cases of proteins lacking the domain of interest. Moreover, any interesting new proteins discovered can readily be added to the input sequence set to compute a new model. This facilitates a flexible strategy of model updating as knowledge accumulates, although a static HMM could, of course, be retrieved at any stage, if desired.

When translated into the HMM formalism, specifying the asymmetric affine gap cost parameters of Program 1, two for insertions and two for deletions, is equivalent to specifying average frequencies and lengths for insertions and deletions [82], uniformly along the HMM. An HMM's free insertion and deletion parameters generally are optimized for the seed alignment provided. Given the sparsity of the seed data concerning the location of gaps, care must be taken to avoid overfitting [108]–[110]. In contrast, we here take the following approach to restricting gap locations based solely upon the BILD scores for columns in the core model, which are data-dense, combined with a few fixed parameters motivated by basic ideas concerning protein structure.

First, we observe that a high BILD score for a column 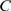 correlates with the column's importance, and indicates the column is unlikely to be deleted, consistent with a general tendency for conserved residues to occur within structural elements crucial for the folding energetics. Let 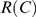 be the mean incremental BILD score for aligning a random residue to 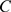. 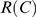 is always negative, and large negative values of 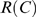 correlate strongly with large positive BILD scores. We set the score (i.e. the log-probability) for extending a deletion through column 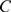 to an empirically chosen multiple 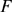 of 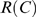. By default, 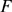 is 2.5. This has the desired effect of penalizing the deletion of columns with high BILD scores. An additional cost 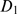 for the existence of a deletion (default: 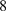 bits) is left uniform throughout the HMM.

Second, we recognize that insertions are relatively unlikely to occur within regions of a protein that show a close clustering of more conserved positions. Let the normalized score 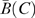 be the BILD score for column 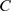 divided by the number of sequences it aligns. We simply disallow insertions anywhere between two columns 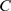 and 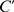 separated by at most one intervening column, when both 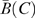 and 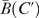 exceed a set threshold 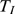 (default: 1 bit). Otherwise, the existence and extension costs, 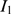 and 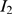 for an insertion are left uniform throughout the HMM with default values of 9.25 and 0.25 bits, as for Program 1. This treatment is motivated by the typical unbroken patterns of local conserved clusters often observed in domain alignments, e.g. the alternating residues of a beta-strand face, or residue pairs within some turn geometries and cap structures. It may be fruitful to extend this insertion model, to conform with observed differences in the frequencies of long and short gaps [89], [111], or to explicitly model the 3–4 spacing of conserved positions commonly seen in alpha-helices.

This simple approach to HMM parameter construction can of course be refined. Nevertheless, it captures central features of the location of insertions and deletions within proteins, without relying upon a preconstructed alignment, or on the relatively small sample of gaps present in a particular data set. Program 2 proceeds identically to Program 1, except that in place of the Erickson-Sellers algorithm with asymmetric affine gap costs, it uses the Viterbi algorithm to find an optimal path through the constructed HMM.

#### Application to Api-AP2 domains

To illustrate how our methods may be applied to typical problems, we consider the sequence-specific DNA recognition domains from the Api-AP2 transcription factor family of apicomplexan parasites. Multiple paralogous Api-AP2 domains in the translated proteomes of *Plasmodium* and *Cryptosporidium* parasites were initially discovered using PSI-BLAST searches by Balaji et al. [112], based on weak similarity to the plant AP2 (APETALA2) transcription factors. These domains also have weak similarity to part of the HNH domain of homing endonucleases [113], [114]. As the principal known sequence-specific DNA binding domains of Apicomplexa, Api-AP2 sequences represent a major lineage-specific gene expansion within the alveolate protists and are currently a topic of intense research [115], [116]. They are believed to function in transcriptional activation crucial for parasite biology and development, and have potential as stage-specific anti-parasitic drug targets, due to the absence of AP2 homologs in the mammalian hosts. It is important to develop a system-level understanding of the Api-AP2 factors, and a prerequisite for this is to discover and annotate the entire complement of Api-AP2 proteins in each of these parasite genomes, possibly beyond the lists obtained from PSI-BLAST and the current HMM-based (Pfam model 00847) searches.

The Api-AP2 family presents a weakly-clustered pattern of amino acid conservation at variable spacing within the typically 50–60 amino acid domain. This profile is present one to approximately six times within otherwise extremely variable protein sequences of typically more than 1000 amino acids. The proteins show little significant homology outside the Api-AP2 regions: there are many low-complexity segments and occasional recognizable domains of other types, but the latter do not show any consistent relationship to the Api-AP2 regions. On the order of 20 to 80 Api-AP2 domains are encoded in each apicomplexan genome. Although relatively small, this domain has typical globular protein structure with a 3-strand beta-sheet packed against an alpha-helix, with several classes of beta-turn, and a longer loop. The more conserved positions occur mainly within the sheet, the helix and beta-turn structural elements. Consequently, multiple alignment profiles tend to show a loosely-patterned clustering of column scores, as is typical of globular domains.

**Figure 1:**
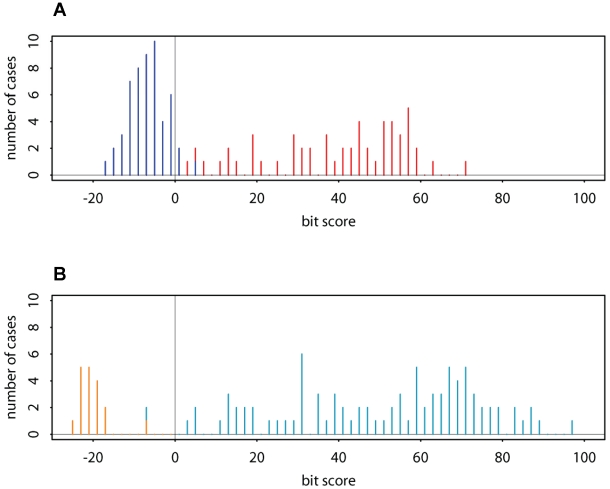
Distributions of bit scores from Api-AP2 domains and negative controls. The histograms in **A** and **B** represent data for both positive and negative cases reported by Program 1 at different intermediate stages of a run. The input file contained 107 amino acid sequences consisting of 54 *T. gondii* proteins with Api-AP2 domain candidates, and 53 random sequences obtained by shuffling the concatenated sequence of 53 of the 54 Api-AP2 proteins and cutting this shuffled string into the original lengths (method of [119]). The Dirichlet mixture prior 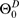 was specified. **A**: Results after the initial Gibbs sampling stage. The ungapped local alignment with optimal aggregate BILD score had width 53. For each sequence, we plot the incremental BILD score, resulting from the addition of a segment from that sequence to the alignment of all the other segments, minus the log of the effective length of that sequence. Scores from the real and random sequences are shown respectively in red and blue. If a prior probability for the existence of a domain in each sequence were specified, segments with scores below a calculated threshold would be rejected. Here, however, the Gibbs sampling step includes one ungapped segment from each of the 107 input sequences in the initial pattern it constructs. **B**: Results after the iterative gapped alignment stage. In each gapped alignment iteration of Program 1, the evolving length-53 pattern is aligned to each input sequence, perhaps multiple times, using a greedy application of the Erickson-Sellers algorithm. Incremental BILD scores are calculated from the current multiple alignment, excluding the sequence to which it is being realigned. Deletions of length 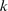 are assigned a score of −8.5−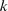 bits, and insertions of length 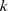 a score of −9.25–0.25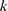 bits. The cost for the existence of a pattern is based on assuming a mean of one instance per sequence, but with uniform probability at all positions of all sequences. In addition, the score for each aligned letter is adjusted slightly to reflect a small cost for not having a gap. At each iteration, the program reports segments with score 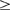
−25 bits, but only segments with positive score are included in the next iteration. We show the data reported for the highest-scoring alignment; at this stage, at least one positively scoring segment derives from each of the 54 real sequences but only 2 segments (each with score less than 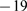 bits) derive from the 53 random sequences. 88 positive-scoring instances of the pattern are found, at least one from each of the real sequences, but none from the random sequences. In addition, 19 instances of the pattern with negative score are found, 2 of which derive from the random sequences. For an aligned segment, a log-odds bit score of 0 indicates an equal probability of being generated by the model implied by the other sequences, or at random by background amino acid frequencies. In **B**, the bars are colored according to the presence (cyan) or absence (brown) of strong sequence matches to the 3 beta-strands and the alpha-helix of the core Api-AP2 structure; the positions of these elements are shown in Figure 3. To qualify for a cyan bar, a sequence was required to contain either identities or high-structural-propensity substitutions that match the strongly conserved amino acids (with column BILD score 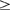
1.5 bits per residue) in the helix and at least 2 of the 3 beta-strands. The fairly clean separation, near 0 bits, of the cyan bars from the others indicates that a positive score is a good criterion for nominating a segment as an Api-AP2 candidate.

Using PSI-BLAST searches of apicomplexan translated genome databases, we collected proteins containing at least one candidate Api-AP2 domain from *Toxoplasma gondii* (53 proteins) and *Plasmodium falciparum* (18 proteins; similar to the set identified by [112]). These sequences were used as input to develop the features of Programs 1 and 2 and to test their ability to construct protein domain profiles and discover additional domains. The results are outlined below, illustrated in Figures 1–3, and presented more fully in Tables S3 and S4 and their caption.

**Figure 2 pcbi-1000852-g002:**
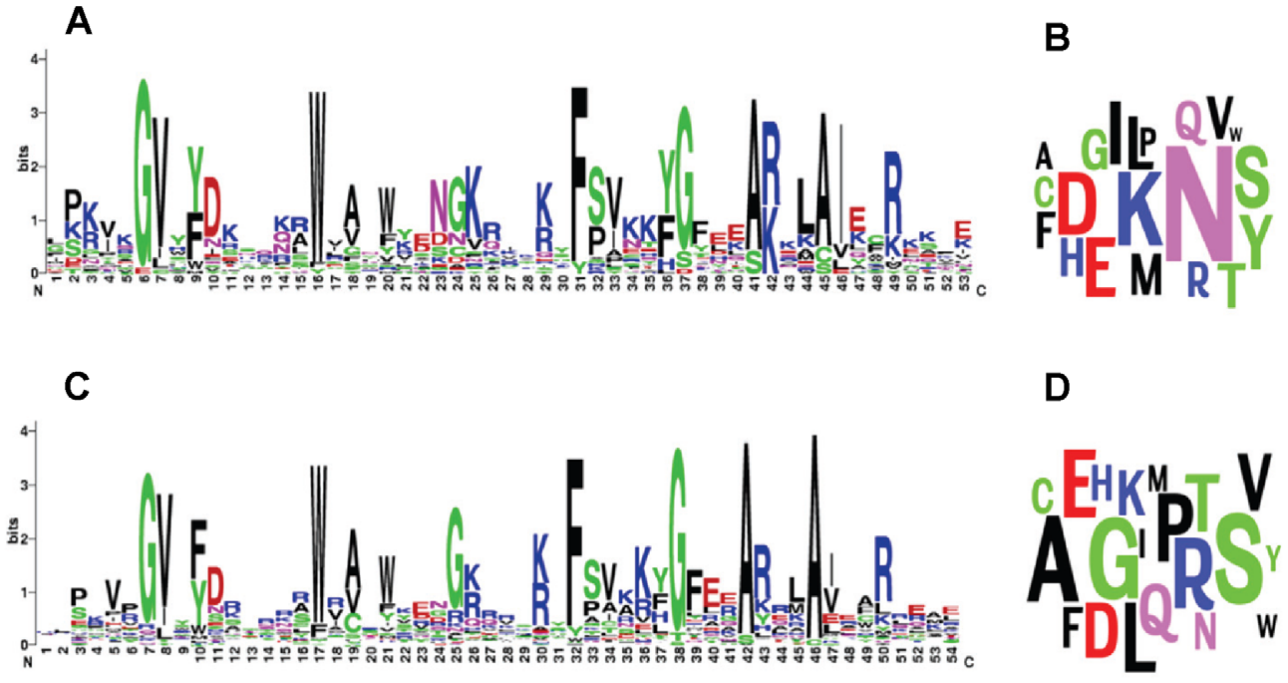
Near-identical Api-AP2 profiles from two parasites with very different background frequencies. For *P. falciparum* (**A**, **B**) and *T. gondii* (**C**, **D**), the logos [120] (http://weblogo.berkeley.edu/) represent the letters aligned in the columns of the core Api-AP2 patterns (**A**, **C**). In the letter clouds (http://www.wordle.net/advanced) (**B**, **D**), the area occupied by each letter indicates the background frequency of an amino acid in the input sequence set (compare Fig. 2.1 of [49]). Colors represent various amino acid classes. For both organisms, Programs 1 or 2, run with Dirichlet mixture priors 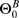, 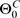 or 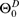, converged on essentially the same 53- to 54-column core models that correspond to these logos. Api-AP2 models and logos almost identical to these were also obtained from other apicomplexan parasites *Cryptosporidium hominis*, *Babesia bovis*, *Theilleria parva*, and from the basal alveolate *Perkinsus marinus*, whereas the distantly related plant AP2 domains and HNH homing endonuclease/integrase domains gave distinct characteristic patterns similar in parts to Api-AP2 (data not shown). Thus, the core structural features of the Api-AP2 domain have been strongly conserved in long-diverged members of the Alveolata, following an ancestral gene expansion, whereas the background amino acid content of these organisms is strikingly different due to genome-wide drift.

**Figure 3:**
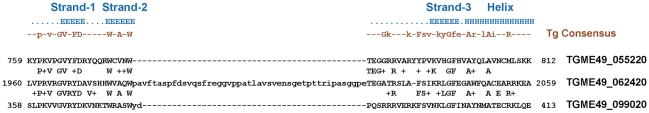
Large insertions in the central loop region of Api-AP2 domains. As a consequence of asymmetric gap costs, Programs 1 and 2 reported several positive Api-AP2 candidates which have long insertions but, in the other parts of the domain, show high-scoring matches to the canonical pattern. Here, the sequence of *T. gondii* protein TGME49_06420, which has a 45 amino acid insertion in the central loop region, is shown aligned with the two most-closely-matching domains of typical length. Program 2, run with Dirichlet mixture prior 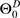 and default parameters, assigned the insertion to the central loop location shown, which avoided the more conserved columns of the secondary structural elements indicated above the sequences. In contrast, Program 1 placed the same inserted residues in three separate locations, two of which would disrupt secondary structure. Moreover, with an established HMM search method [80] (http://hmmer.janelia.org/), only the right end alignment of this TGME49_06420 domain was found, but with a negative score well below the rejection threshold. Structural assignments E (beta-strand) and H (alpha-helix) are based on homologous experimental structures [121], [122] (PDB codes 2gcc,3gcc,3igm).

To explore how Programs 1 and 2 can tolerate negative cases, lacking the domain of interest, we spiked the Api-AP2 input sets with various proportions of (a) random sequences constructed by shuffling input sequences, or (b) real sequences lacking annotated conserved patterns, or (c) sequences that shared a conserved domain unrelated to Api-AP2. Spikes of types (a) and (b) comprising half of the total input sequences did not affect the final Api-AP2 models: the random and unrelated patterns in the spike sequences were all rejected (Figure 1) during or after the initial ungapped Gibbs sampling step, and this step runs faster if one specifies a prior expectation that a fraction of the input sequences do not contain the pattern of interest. If the Gibbs sampling stage of either Program 1 or 2 is run with a prior expectation that 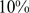 of the input sequences do not contain an instance of the pattern, then all the random sequences and five of the Api-AP2 sequences are initially excluded, but subsequent gapped alignment steps recover segments from the initially rejected Api-AP2 sequences. The final result is the same whether or not sequences are excluded in the initial stage. With some spikes of type (c), the Gibbs sampling step converged on the competing domain instead of the Api-AP2 pattern. This suggests that input sequence domain parsing, e.g. by methods in [117], [118], may sometimes be beneficial.

The amino acid frequencies observed within the core Api-AP2 model were strikingly similar for *Plasmodium* and *Toxoplasma* (Figure 2), consistent with an evolutionary expansion of this family from a single ancestral gene within the Alveolata, as proposed by [112]. Present day parasite lineages have evolved strikingly different codon and background amino acid content arising from genomic drift, e.g. in the very AT-rich *Plasmodium* and the more GC-enriched *Toxoplasma*. This contrast in background frequencies (Figure 2) demonstrates the value of log-odds scores for identifying a subtle pattern in very different sequence contexts.

The Api-AP2 pattern is present more than once in many of the input sequences as is often the case with eukaryotic multidomain proteins, potentially enabling these transcription factors to recognize combinations of DNA sites. The greedy algorithm included in Programs 1 and 2 allows such repeated domains to be identified. A total of 89 domains were found within the initial 53 *Toxoplasma* input sequences. Only 2 domains had borderline scores and may be candidates for classification as degenerate pseudo-domains. As shown in Table S3, repeats in the same protein can be very diverse in sequence. Programs 1 and 2 found several repeated domains additional to those reported in searches with Pfam model 00847, including some that differ from the canonical domain length by relatively long insertions in the central loop region (Figure 3, Table S3).

We conducted further searches of the *Toxoplasma* and *Plasmodium* databases based on the core Api-AP2 alignment obtained from Programs 1 and 2. These revealed new candidate proteins with Api-AP2 domains (Tables S3 and S4), not found with Pfam model 00847, some of which also show long loop insertions, but are otherwise strongly similar to the canonical Api-AP2 domain sequence (Figure 3). Including these new Api-AP2 cases, we have identified a total of 68 proteins (103 domains) for *Toxoplasma* and 29 proteins (50 domains) for *Plasmodium* (Tables S3 and S4).

It is not yet known if Api-AP2 domains with long insertions are active in DNA binding and transcriptional control, or whether any are inactive pseudo-domains, or are artifacts from errors in gene modeling. However, their occurrence illustrates that a relatively small minority of members of a domain family may contain long insertions, a general feature of protein evolution. Experimental studies confirm that many such long insertions, when artificially engineered into structural loops, have surprisingly low costs for the free energy of folding and little effect on the functional interactions of the proteins [90], [91]. Thus, both the observed occurrence and the statistical thermodynamics of long insertions justify our treatment, described above, using asymmetric affine gap costs.

## Discussion

We have described a natural generalization of log-odds substitution scores for pairwise alignments to substitution scores for multiple alignment columns. Multiple alignment log-odds scores probably are best derived using a Bayesian approach, yielding what we have called BILD scores. Log-odds scores imply scores for aligning multiple alignment columns to one another, or for aligning multiple alignment columns to single sequences, and it was in this latter context that the Bayesian approach was first formulated by Brown 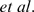 [31]. In conjunction with the Minimum Description Length Principle, log-odds scores provide a means for determining the proper width or extent of a local multiple alignment, and for deciding whether a segment should be included in the alignment. They may also be used to cluster a set of related segments into subclasses; see Text S4 and [62], [71], [72].

One may compute rapidly the BILD score for a multiple alignment column, as well as the new score that results from the addition or subtraction of a single letter. This permits BILD scores to be used practically in Gibbs-sampling local multiple alignment programs. They can improve the performance of such programs, and remove the need for specifying the width of a pattern sought.

The proper description of protein domains in most cases requires a provision for gaps. We have implemented two relatively simple programs for extending a core ungapped pattern or profile to a gapped local multiple alignment. There are several key elements to our approach. First, the initial maximization of aggregate BILD scores using Gibbs sampling yields a core pattern and pattern length for further refinement. Second, the semi-global alignment of this pattern to the input sequences recognizes the importance of complete occurrences of the pattern. Third, the use of asymmetric affine gap costs (Program 1) recognizes that, with respect to the core pattern, long deletions generally are much more deleterious than long insertions. The placement of gaps can be refined using position-specific gap costs derived from column BILD scores (Program 2). Fourth, greedy alignment allows multiple instances of a pattern to be found within a single sequence. In conjunction with length-dependent gap costs, it discourages alignments spanning more than one instance of a pattern, but can still uncover long insertions. Fifth, iteration permits the core model to be refined, improving the discrimination of true relationships from chance similarities. This strategy, informed by considerations of protein structure, has proved a rapid and effective method for delineating protein families. Although our programs were developed only for research purposes, with the limited goal of testing the impact of BILD scores, their code is available upon request.

We have sought here primarily to describe the construction and potential uses of log-odds scores in the multiple alignment context. However, many avenues for further research, involving the development and benchmarking of complete multiple alignment programs, remain. To what extent can BILD scores improve the accuracy of profile-profile comparison programs? How does Erickson-Sellers semi-global alignment [92], with uniform asymmetric affine gap costs, compare to HMM [80], [81] and other methods [6] in recognizing related sequence in database searches? We look forward to investigating some of these questions.

## Supporting Information

Text S1 MELD Scores
(0.05 MB PDF)

Text S2 The Mean Relative Entropy of Dirichlet Mixtures
(0.05 MB PDF)

Text S3 The MDL Principle and Local Alignment Statistics
(0.04 MB PDF)

Text S4 The MDL Principle and the Clustering of Multiple Alignments
(0.05 MB PDF)

Text S5 Gibbs Sampling Algorithms and HTH Proteins
(0.05 MB PDF)

Table S1 Helix-turn-helix proteins.
(0.02 MB PDF)

Table S2 Number of sequences misaligned by Gibbs sampling programs. Sequence sets supplied to the BILD and Wadsworth samplers consist of the first *M* sequences listed in Table S1. For each sequence set, the BILD sampler determines an optimal motif width *W*. Both BILD and Wadsworth samplers optimize contiguous motifs of widths *W*, 17, 21 and 25. The number of sequences misaligned by the Wadsworth sampler are given in the table without parentheses; the number misaligned by the BILD sampler within parentheses.
(0.02 MB PDF)

Table S3 Tables S3 and S4 show Api-AP2 domains and bit scores reported by Programs 1 and 2 for *Toxoplasma gondii* (Table S3) and *Plasmodium falciparum* (Table S4). Also shown are the bit scores obtained using HMMsearch database searches [Eddy SR (1998) *Bioinformatics* 14: 755–763] seeded with aligned Api-AP2 domains and with the current Pfam AP2 model number 00847 (http://pfam.sanger.ac.uk/family?entry=PF00847). Programs 1 and 2 were run with the Dirichlet mixture prior and default parameters described in the Results section and Figures 1 and 3. As input, we collected 68 amino acid sequences from *T. gondii* and 29 from *P. falciparum*, based on inspection of low-threshold PSI-BLAST and HMMsearch searches of the parasite genomic translation databases of ToxoDB [Gajria *et al.* (2008) *Nucleic Acids Res* 36: D553–556] and PlasmoDB [Aurrecoechea *et al.* (2009) *Nucleic Acids Res* 37: D539–543] (http://eupathdb.org/eupathdb/). These database searches were seeded with earlier alignments produced (as described in the Results section and Figure 1 legend) from more preliminary sets of 54 *T. gondii* and 18 *P. falciparum* sequences. We anticipated that the larger sets of input sequences might include some false positives; however, the final evolved models included at least one positive score from each of the 68 and 29 sequences, totaling 103 Api-AP2 domain candidates for *T. gondii* and 50 for *P. falciparum*. The corresponding core domain alignments assigned by Program 2 are shown, denoted respectively ‘Tg-core’ (with 53 columns in the evolved model plus 2 adjacent positively-scoring columns added from the left-flank) and ‘Pf-core’ (with 53 columns) respectively. These patterns exclude any insertions in individual sequences: the number of inserted residues is shown in a separate column. All of these domains have positive bit scores with Programs 1 and 2, except for the special case of domain 1.7, Table S3, which has been added manually to the *T. gondii* alignment. This domain is notable because of its occurrence within a multi-Api-AP2 protein and its strong match to the canonical 53-column pattern; however, it also has an unusually long insertion of 66 amino acids (assigned to the central loop by Program 2), the cost of which results in an overall negative score. The Tg-core (excluding domain 1.7) and the Pf-core alignments shown in Tables S3 and S4 were used as seed alignments for further analysis with HMMER version 2.3.2 (http://hmmer.janelia.org). HMMbuild and HMMcalibrate were used with default parameters to construct HMMs and calibrate their E-value distributions, and HMMsearch was used with a permissive E-value threshold of 100 to search the parasite genomic translation databases against these HMMs. These searches gave positive bit scores for the domains used for HMM construction (except domain 1.8, which was not reported, Table S3), as shown in the columns headed ‘bits (HMMsearch, Tg-core seed)’ and ‘bits (HMMsearch, Pf-core seed)’. In some cases, HMMsearch alignments encompassed only part of the Api-AP2 pattern, either to the left or right of the central loop, denoted, respectively, ‘LH only’ and ‘RH only’ in comments columns. Note that all of the positively scoring sequences reported were present in the seed alignment, and no new Api-AP2 domain candidates were found in these HMMsearch database searches. HMMsearch scans of the same databases were also seeded by Pfam model 00847 (converted to a version 2.3.2 HMM with HMMbuild and HMMcalibrate as described above). The resulting bit scores are given in the columns headed ‘bits (HMMsearch, Pfam00847 seed)’. The Pfam00847 model seed alignment contains both plant and apicomplexan AP2 domains, including some from *P. falciparum* but none from *T. gondii*. Consequently, the matches of Pfam00847 to the Api-AP2 domains are generally weaker than the matches obtained with the more specific models from Program 2 Api-AP2 core alignments, resulting in substantially lower bit scores. Several domain candidates (highlighted in color), 10 from *T. gondii* and 4 from *P. falciparum*, were not reported by the Pfam00847 HMMsearch above the E-value 100 threshold, and others (9 and 3 respectively) were given negative scores (and non-significant E-values). These low scores reflect misalignments (e.g. missed long insertions) in some cases. In other cases, limited deviations from the canonical conserved patterns occur, commonly in the first beta-strand. However, such deviant residues appear to be structurally compatible with the domain, with beta-strand-favoring propensities in most cases, suggesting that these examples may be authentic but non-canonical Api-AP2 domains. HMMER methodology is capable of identifying and aligning such domains if they are included in the seed alignment, as shown by the bit scores given by the Tg-core and Pf-core seeded searches. Indeed, the *T. gondii* domain 62.1 (TGME49_062420), which is the example with a 45/46 amino acid insertion shown in Figure 3, obtains a positive bit score with HMMsearch (and 46 inserted residues) when it is included in the Tg-core seed alignment (as in Table S3) but a negative score and only a partial alignment otherwise, as indicated in Figure 3 legend. In several cases, HMMsearch alignments report different gap positions from Program 2, mostly with shorter insertions. In the case of domain 66.1 (Table S3) the alignment produced by HMMsearch appears to be more compatible with beta-strand propensities than the Program 2 alignment shown in Table S3, whereas in 6 other cases, the HMMsearch alignment appears more disruptive of secondary structure. This observation supports the potential benefit of incorporating secondary structure prediction into an HMM-based domain recognition strategy, as proposed by Won *et al.* [(2007) *BMC Bioinformatics* 8: 357]. Overall, the HMMsearch results shown in these Tables, compared with the Programs 1 and 2 output, show many more similarities than differences: the two approaches can achieve very similar results with appropriate inputs. Our examples also illustrate how the relatively simple BILD score based approaches, by reducing the strict dependence on seed alignments, might facilitate more automated processes for the discovery and reporting of protein domain families and more flexible updating strategies.
(0.05 MB XLS)

Table S4 Api-AP2 domains and bit scores reported by Programs 1 and 2 for *Plasmodium falciparum*. For more details please see caption to Table S3.
(0.03 MB XLS)
