# Supplementary material for: The Construction and Use of Log-Odds Substitution Scores for Multiple Sequence Alignment
Source: PLoS Comput Biol. 2010 Jul 15;6(7):e1000852. doi: 10.1371/journal.pcbi.1000852 (PMC2904766; doi:10.1371/journal.pcbi.1000852)
Supplement: Text S1 — MELD Scores (0.05 MB PDF) [file pcbi.1000852.s001.pdf]

Text S1: MELD Scores

The feature of multiple alignment column scores essential to the applications described in this paper is their explicit construction as log-odds scores, which in turn requires the derivation of target frequencies $Q(\vec{x})$ for multiple alignment columns. The Bayesian formalism underpinning BILD scores is not the only rational basis for constructing such target frequencies. As we describe here, values for $Q(\vec{x})$ can also be derived from any pairwise substitution matrix.

Any local alignment substitution matrix is implicitly of log-odds form [1], and can be adjusted to a "valid" matrix that is appropriate for comparing sequences with the background frequencies p_i [2, 3]. For such a valid matrix, $s_{i,j} = [\ln(q_{i,j}/p_i p_j)]/\lambda$, with $p_i = \sum_{j=1}^{L} q_{i,j}$. Then, for related letters, we have $q_{i,j} = p_i p_j \; e^{\lambda s_{i,j}}$, and can also write $q_{i,j} = p_i \mathrm{Prob}(j|i)$. Combining these two equations yields

$$\mathrm{Prob}(j|i) = p_j \; e^{\lambda s_{i,j}} \; . \qquad (1)$$

Imagine assigning the letters of $\vec{x}$ to the leaves of an evolutionary tree, for simplicity taken to be a star with equal branch lengths. By summing over the possible unobserved letters at the central node, weighted by their background probabilities p_i, one gets

$$Q(\vec{x}) = \sum_{i=1}^{L} p_i \prod_{j=1}^{M} \mathrm{Prob}(x_j|i) \; . \qquad (2)$$

We will call column scores constructed in this manner "Mean Evolutionary-tree Log-odds" or MELD scores.

If the original substitution scores $s_{i,j}$ correspond to an evolutionary distance D then, for the $Q(\vec{x})$ to which equations (1) and (2) give rise, the implied evolutionary distance between any two letters of $\vec{x}$ is $2D$. Thus if the $s_{i,j}$ are those of a PAM-100 substitution matrix [4,5], the $Q(\vec{x})$ constructed when $M = 2$ will be that of a PAM-200 matrix. It is possible to generalize MELD scores to trees other than a star, and to means taken over various possible evolutionary trees with specified prior probabilities. This, however, quickly becomes unwieldy, so we will consider only MELD scores confined to a star. The sometime inappropriateness of such an evolutionary model can be mitigated by the use of sequence weights, with each $\mathrm{Prob}(x_j|i)$ term in equation (2) raised to the power of the weight of sequence j, and with a corresponding treatment for $P(\vec{x})$.

For the alignment of a large number M of sequences, MELD scores have a major theoretical disadvantage to BILD scores. Under the Bayesian formalism, for large M, the probabilities predicted for a new letter added to a particular column converge to the observed letter frequencies. Thus the observation of only leucines in a protein position will for large M reduce the predicted probability for any other residue a to near zero. However, with MELD scores constructed, e.g., from the PAM-100 $s_{i,j}$, all that occurs as M grows is the gradual change from the PAM-200 to the PAM-100 target frequency for the alignment of a to leucine.

The reason we introduce MELD scores at all is that they may have advantages for certain applications. Standard substitution matrices are non-optimal for the comparison of sequences with non-standard background letter frequencies [2], and it is possible to adjust a standard symmetrical matrix into an asymmetric one suitable for the comparison of sequences with differing

background frequencies [2,3]. This strategy may be extended to MELD scores for the comparison of three or more sequences, each with its own distinct background frequencies. The letter frequencies at the central node can be taken to be the average, perhaps weighted, of the letter frequencies for the various input sequences. Distinct asymmetric substitution scores can then be constructed for the comparison of this central node to each of the input sequences. Distinct values of $\text{Prob}(j|i)$ are thus derived, through equation (1), for the alignment of the letter i at the central node to the letter j in each of these sequences. This yields asymmetric MELD scores adapted to the particular background frequencies of the sequences being compared. Such scores may be of value for the alignment of a small number of sequences that are related but nevertheless have very divergent background frequencies.

The $Q(\vec{x})$ used in the construction of BILD scores can be adjusted for sequences with non-standard composition, using a generalization of the approach for pairwise target frequencies [2,3]. However, this involves an optimization in an $L^M - 1$ dimensional space. For proteins, with $L = 20$, this may be impractical for $M > 3$.

References

1. Karlin S, Altschul SF (1990) Methods for assessing the statistical significance of molecular sequence features by using general scoring schemes. Proc Natl Acad Sci USA 87: 2264–2268.

2. Yu YK, Wootton JC, Altschul SF (2003) The compositional adjustment of amino acid substitution matrices. Proc Natl Acad Sci USA 100: 15688-15693.

3. Yu YK, Altschul SF (2005) The construction of amino acid substitution matrices for the comparison of proteins with non-standard compositions. Bioinformatics 21: 902-911.

4. Dayhoff MO, Schwartz RM, Orcutt BC (1978) A model of evolutionary change in proteins. In: Dayhoff MO, editor, Atlas of Protein Sequence and Structure, Washington, DC: Natl. Biomed. Res. Found., volume 5, suppl. 3. pp. 345–352.

5. Schwartz RM, Dayhoff MO (1978) Matrices for detecting distant relationships. In: Dayhoff MO, editor, Atlas of Protein Sequence and Structure, Washington, DC: Natl. Biomed. Res. Found., volume 5, suppl. 3. pp. 353–358.
