# Supplementary material for: The Construction and Use of Log-Odds Substitution Scores for Multiple Sequence Alignment
Source: PLoS Comput Biol. 2010 Jul 15;6(7):e1000852. doi: 10.1371/journal.pcbi.1000852 (PMC2904766; doi:10.1371/journal.pcbi.1000852)
Supplement: Text S2 — The Mean Relative Entropy of Dirichlet Mixtures (0.05 MB PDF) [file pcbi.1000852.s002.pdf]

Text S2: The Mean Relative Entropy of Dirichlet Mixtures

We are first interested in calculating $\hat{\mathcal{D}}$, the mean value of the relative entropy $\sum_i q_i \ln(q_i/p_i)$ when the probability density of $\vec{q}$ follows a Dirichlet distribution. (Note that throughout this appendix we use natural logarithms, and all relative entropies are therefore expressed in nats rather than bits.) Specifically, as stated in the main text, $\rho(\vec{q}) \equiv \mathcal{Z} \prod_j q_j^{\alpha_j - 1}$, where $\mathcal{Z} = \Gamma(\alpha^*)/\prod_j \Gamma(\alpha_j)$. The domain of $\vec{q}$ is the simplex $\mathcal{P}$ defined by $0 \le q_i \le 1$ and $\sum_i q_i = 1$. The scalar $\mathcal{Z}$ arises from the fact that

$$\int_{\vec{q}\in\mathcal{P}} \prod_j q_j^{\alpha_j-1}\, d\vec{q} = \frac{\prod_j \Gamma(\alpha_j)}{\Gamma(\alpha^*)} \; . \tag{1}$$

The mean value of the ith component of the relative entropy is given by

$$\begin{aligned}
\mathcal{Z} \int_{\vec{q}\in\mathcal{P}} \left[q_i \ln \frac{q_i}{p_i} \right] \prod_j q_j^{\alpha_j-1}\, d\vec{q} &= \mathcal{Z} \int_{\vec{q}\in\mathcal{P}} [\ln q_i - \ln p_i]\, q_i^{\alpha_i} \prod_{j\neq i} q_j^{\alpha_j-1}\, d\vec{q} \\
&= \mathcal{Z}\, [\partial_{\alpha_i} - \ln p_i] \int_{\vec{q}\in\mathcal{P}} q_i^{\alpha_i} \prod_{j\neq i} q_j^{\alpha_j-1}\, d\vec{q} = \mathcal{Z}\, [\partial_{\alpha_i} - \ln p_i]\, \frac{\Gamma(\alpha_i+1)\prod_{j\neq i}\Gamma(\alpha_j)}{\Gamma(\alpha^*+1)} \\
&= \mathcal{Z}\, [\psi(\alpha_i+1) - \ln p_i - \psi(\alpha^*+1)]\, \frac{\Gamma(\alpha_i+1)\prod_{j\neq i}\Gamma(\alpha_j)}{\Gamma(\alpha^*+1)} \\
&= [\psi(\alpha_i+1) - \ln p_i - \psi(\alpha^*+1)]\, \frac{\alpha_i}{\alpha^*} \; ,
\end{aligned} \tag{2}$$

where $\psi(x) \equiv (d/dx) \ln \Gamma(x)$. When we sum (2) over all i, noting that $\sum_i \alpha_i = \alpha^*$, we find that

$$\hat{\mathcal{D}} = \mathcal{Z} \int_{\vec{q}\in\mathcal{P}} \left[\sum_i q_i \ln \frac{q_i}{p_i} \right] \prod_j q_j^{\alpha_j-1}\, d\vec{q} = \left\{ \sum_i [\psi(\alpha_i+1) - \ln p_i]\, \frac{\alpha_i}{\alpha^*} \right\} - \psi(\alpha^*+1) \; . \tag{3}$$

For a Dirichlet mixture, we have $\hat{\mathcal{D}} = \sum_k m_k \hat{\mathcal{D}}_k$, where $\hat{\mathcal{D}}_k$ is the mean relative entropy of the kth component Dirichlet distribution.

A simple numerical formula for the digamma function $\psi(x)$ is

$$\psi(x+1) = -\gamma + \sum_{k=1}^{\infty} \left(\frac{1}{k} - \frac{1}{x+k} \right) = -\gamma + \sum_{k=1}^{\infty} \frac{x}{k(x+k)} \; , \tag{4}$$

where γ is the Euler's constant $0.5772\ldots$. Because eq. (3) involves a difference of digamma functions, the γ term in eq. (4) is irrelevant.
