# Supplementary material for: The Construction and Use of Log-Odds Substitution Scores for Multiple Sequence Alignment
Source: PLoS Comput Biol. 2010 Jul 15;6(7):e1000852. doi: 10.1371/journal.pcbi.1000852 (PMC2904766; doi:10.1371/journal.pcbi.1000852)
Supplement: Text S3 — The MDL Principle and Local Alignment Statistics (0.04 MB PDF) [file pcbi.1000852.s003.pdf]

Text S3: The MDL Principle and Local Alignment Statistics

It is instructive to compare equation (12) of the main text to the E-value formula for ungapped local alignments with score S bits:

$$E(S) = K \left(\prod_{i=1}^{M} N_i \right) 2^{-S}, \tag{1}$$

where K is a constant dependent upon the specific scoring system [1]. The E-value is less than 1 whenever S exceeds $\sum_{i=1}^{M} \log N_i + \log K$. This is the same condition as that for preferring T_1, except with $\log K$ replacing L_W. If we wish to equate these conditions, however, we have a seeming problem, because K is always less than 1, implying $\log K$ is negative, whereas L_W is positive. The problem can be resolved by observing that $L(T_1)$ should represent the description length of only "effectively independent" parameters [2]. In other words, when it is difficult to choose among various values for a theory's parameters based upon the data, the effective parameter space is correspondingly smaller than it might appear. This is the case here, because it is frequently difficult for the data to strongly prefer one alignment position vector $\vec{s}$ to another with all coordinates increased or decreased by a small integral constant, and it is also frequently difficult for the data to strongly prefer one value of W to another. Accordingly, we postulate that these considerations imply that the term L_W in equation (12) of the main text should be replaced by $\log K$, with K derived from the statistical theory [1]. For both DNA and protein sequence comparison, using BILD scores with standard Dirichlet priors, calculation shows that $\log K$ tends to decrease as the number M of sequences increases, but rarely by more than about 0.1 bit per sequence (data omitted).

References

1. Karlin S, Altschul SF (1990) Methods for assessing the statistical significance of molecular sequence features by using general scoring schemes. Proc Natl Acad Sci USA 87: 2264–2268.

2. Grünwald PD (2007) The Minimum Description Length Principle. Cambridge, MA: MIT Press.
