# Supplementary material for: The Construction and Use of Log-Odds Substitution Scores for Multiple Sequence Alignment
Source: PLoS Comput Biol. 2010 Jul 15;6(7):e1000852. doi: 10.1371/journal.pcbi.1000852 (PMC2904766; doi:10.1371/journal.pcbi.1000852)
Supplement: Text S4 — The MDL Principle and the Clustering of Multiple Alignments (0.05 MB PDF) [file pcbi.1000852.s004.pdf]

Text S4: The MDL Principle and the Clustering of Multiple Alignments

Once one has constructed or been given a multiple alignment of related sequences, it is sometimes desirable to divide it into several subalignments, perhaps representing subfamilies. The MDL principle suggests how this may best be done. Consider a fixed alignment (assumed to be accurate) of related segments from M sequences. This alignment of M segments may be divided into anywhere from 1 to M classes, and the division into i classes may be assigned a prior probability π_i. Usually there is no reason to favor one number of classes over another, and the π_i may be set to a uniform value of $1/M$, but this is not necessary and one may for example prefer a geometric distribution. Assigning the sequences to G classes requires M indicator parameters I_i, each with a range 1 to G. The description length of these parameters is $M \log G - \log G\,!$ bits, where the second term recognizes that the labels on the classes are arbitrary and may be permuted. It is possible for this increase in the description length of the theory to be offset by a decrease in the description length of the data, which may arise when a single log-odds score is replaced by several. Thus we should choose G and the M associated indicator parameters I_i to minimize

$$DL_G = M \log G - \log G\,! - \log \pi_G - \sum_{i=1}^{G} S_i \ , \qquad (1)$$

where S_i is the log-odds score of class i. For each G beginning with 2, one may use a Gibbs sampling algorithm to optimize the I_i. Note that here, the sampler is choosing only class assignment, not realigning the sequences. For uniform π_i, we have found that as G increases, once DL_G begins to decrease, it almost never increases again.

If the segments are weighted to reflect correlations, we argue that M in formula (1) should represent the effective rather than the actual number of segments, because the I_i will not be independent. For example, consider the extreme case in which two identical segments A and B are each assigned weight 1/2, but all other segments are assigned weight 1. We would like the minimization of (1) to produce the same result it would if segment B were dropped, and segment A were given weight 1. Assuming segments A and B are assigned to the same class, the log-odds score for this class will, by construction, be the same as if only sequence A with weight 1 had been included. However, unless M in formula (1) is defined to reflect sequence weights, DL_G will vary for the two cases, perhaps thereby leading to different optimal values of G.

With several differences, an analog of this procedure has been proposed previously [1, 2]. We consider these differences, which touch upon all four terms of equation (1). (i) It was not previously suggested that M should reflect segment weights, as discussed above. (ii) The term $-\log G\,!$ was previously omitted; this can be seen as a minor error in analysis. (iii) The term $-\log \pi_G$ was previously omitted. This new term is a minor generalization, and has no effect if the π_i are chosen to be uniform. (iv) Using our notation, the previous analysis omitted the $P(\vec{x})$ terms in equation (1) from its analog of the S_i in equation (1). When the alignment is fixed for all G, this is a distinction without a difference, because the inclusion of the $P(\vec{x})$ terms effects all the DL_G equally. However, as we discuss below, one may allow the alignment to vary with G, in which case it is necessary to include the $P(\vec{x})$. (v) Some previous approaches choose the I_i through the construction of an evolutionary tree relating the segments, and cutting it at varying depths. This ensures that the classes chosen for one G can always be nested within

those chosen for a smaller G'. However, there is no need for this restriction, and we have found that it is frequently violated by optimal classes for varying Gs. For many applications, the construction of a tree relating the classes for varying Gs may bring added value, but it is not always useful, well defined, or indeed appropriate. A Gibbs sampling procedure for optimizing the I_i is heuristic, as is the tree construction procedure. However, it is less likely to be trapped in suboptimal solutions, and should be of sufficient speed for most practical problems. More sophisticated sampling algorithms are also possible [3].

The optimal extent of local optimal alignment may depend upon just which sequences are included. However, given a fixed multiple alignment, it is possible to optimize the starting and stopping positions, and implied width W, of the alignment separately for each G. This requires the fitting of additional parameters, but so long as there is but one optimization for each G, applicable to all classes, the description lengths of the new parameters do not vary with G, and may therefore be effectively ignored.

References

1. Sjölander K (1998) Phylogenetic inference in protein superfamilies: analysis of SH2 domains. In: Glasgow J, Littlejohn T, Major F, Lathrop R, Sankoff D, et al., editors, Proc. Sixth Int. Conf. on Intelligent System for Mol. Biol. Menlo Park, CA: AAAI Press, pp. 165-174.

2. Brown DP, Krishnamurthy N, Sjölander K (2007) Automated protein subfamily identification and classification. PLoS Comput Biol 3: e160.

3. Brown DP (2008) Efficient functional clustering of protein sequences using the Dirichlet process. Bioinformatics 24: 1765–1771.
