# Supplementary material for: The Construction and Use of Log-Odds Substitution Scores for Multiple Sequence Alignment
Source: PLoS Comput Biol. 2010 Jul 15;6(7):e1000852. doi: 10.1371/journal.pcbi.1000852 (PMC2904766; doi:10.1371/journal.pcbi.1000852)
Supplement: Text S5 — Gibbs Sampling Algorithms and HTH Proteins (0.05 MB PDF) [file pcbi.1000852.s005.pdf]

Text S5: Gibbs Sampling Algorithms and HTH Proteins

Gibbs sampling for local multiple sequence alignment was introduced by Lawrence *et al.* [1], and has since undergone substantial development by Lawrence and colleagues [2,3]. Given a set of input sequences and a specified pattern width W, the algorithm seeks a segment of length W within each sequence so that the aligned segments maximize a specified objective function. To start, a provisional starting position for a length-W segment is chosen randomly within each sequence and the algorithm then proceeds iteratively as follows. The segment from one sequence S is dropped from the implied alignment, and position-specific multinomial frequencies are estimated from the remaining segments. Then, for each feasible position i within sequence S, probabilities Q_i and P_i are calculated for generating the length-W segment starting at i, using respectively the position-specific multinomial and the background letter frequencies. Finally, weighted by the ratios Q_i/P_i, a random segment within S is selected and added to the alignment, and the process repeats by dropping the segment from a different sequence.

A public web site, http://bayesweb.wadsworth.org/gibbs/gibbs.html, implementing such a Gibbs motif sampler is available from the New York State Department of Health's Wadsworth Center. The Wadsworth sampler constructs motif models for either protein or DNA sequences, attempting to optimize a relative-entropy-based objective function [1–3]. In the context of Gibbs sampling, this is algorithmically equivalent to employing a single Dirichlet prior to construct profile-sequence alignment scores. The effective default Dirichlet parameters used by the Wadsworth sampler are $\alpha_j = 0.1(M-1)p_j$.

The innovations proposed here are the adoption of Dirichlet mixture-based profile-sequence scores for protein comparison, and the dynamic selection of pattern width through the optimization of BILD scores. We have implemented a simple Gibbs sampling program, which we will call the BILD sampler, to test these ideas in comparison with the Wadsworth sampler. The Dirichlet mixture priors described in Table 1 of the main text with $\mathcal{D}_2$ most appropriate for distantly related proteins are Θ_0^C and Θ_0^D. The example described here uses Θ_0^D, but roughly equivalent results are obtained with Θ_0^C.

Given a fixed number of columns within a motif, the Wadsworth sampler allows the motif's columns to be discontiguous. The MDL principle can be applied to discontiguous patterns, but we simplify our present study by considering only contiguous patterns, with both the BILD and Wadsworth samplers. In our implementation, the BILD sampler optimizes an ungapped pattern for a fixed width. It then determines whether changing the boundaries of the pattern would increase the BILD score. If so, the sampler is rerun, using the new pattern width.

To construct sequence sets of varying difficulty, we began with the thirty helix-turn-helix proteins from [1], in the random order given in Table S3, and considered subsets consisting of the first M sequences. Neither the BILD nor Wadsworth sampler was able to align any sequences correctly for M less than 4. (The "gold standard" for proper alignment is based on structural evidence, as described in [1].) For M from 4 to 30, we ran the BILD and Wadsworth samplers for fixed pattern widths 17, 21 and 25. We also allowed the BILD sampler to choose dynamically what it calculated to be the optimal motif width W, as described above, and we then ran both the BILD and Wadsworth samplers with this width. For all sequence sets and motif widths, the number of input sequences misaligned are shown in Table S4, with the results for the BILD

sampler appearing within parentheses.

Of the 108 (sequence set, motif width) pairs considered, the BILD sampler misaligned fewer sequences than the Wadsworth sampler 65 times, and more 6 times. Omitting the widths W chosen dynamically by the BILD sampler, it performed better 48 and worse 5 times. For any of the fixed widths, the BILD sampler makes alignment errors on fewer sequence sets than the Wadsworth sampler, and makes fewer aggregate errors over all sequence sets. Of course, one does not in general know *a priori* the proper width to specify. The BILD sampler does a reasonable job of selecting a width to use, avoiding any misalignment errors for test sets with $M > 10$, although it could have done better by uniformly using width 21.

References

1. Lawrence CE, Altschul SF, Boguski MS, Liu JS, Neuwald AF, et al. (1993) Detecting subtle sequence signals: A Gibbs sampling strategy for multiple alignment. Science 262: 208-214.

2. Liu JS, Neuwald AF, Lawrence CE (1995) Bayesian models for multiple local sequence alignment and Gibbs sampling strategies. J Amer Stat Assoc 90: 1156-1170.

3. Thompson W, Rouchka EC, Lawrence CE (2003) Gibbs recursive sampler: finding transcription factor binding sites. Nucleic Acids Res 31: 3580-3585.
