# Supplementary material for: The Construction and Use of Log-Odds Substitution Scores for Multiple Sequence Alignment
Source: PLoS Comput Biol. 2010 Jul 15;6(7):e1000852. doi: 10.1371/journal.pcbi.1000852 (PMC2904766; doi:10.1371/journal.pcbi.1000852)
Supplement: Table S1 — Helix-turn-helix proteins. (0.02 MB PDF) [file pcbi.1000852.s006.pdf]

Table S1. Helix-turn-helix proteins.

	Protein or gene name	Swiss-Prot accession number
1	MATa1	P01366
2	Sigma-37	P06574
3	Pin	P03014
4	P22 Cro	P09964
5	GalR	P03024
6	MerD	P08654
7	TetR	P04483
8	P22 c1	P03041
9	TrpR	P0A881
10	Antennapedia (Antp)	P02833
11	NahR	P10183
12	Fnr	P0A9E5
13	Crp (CAP)	P0ACJ8
14	AraC	P0A9E0
15	LexA	P0A7C2
16	NtrC (K.p.)	P03029
17	Lambda Cro	P03040
18	DeoR	P0ACK5
19	LacI	P03023
20	Sigma-32 (rpoH,htpR)	P0AGB3
21	SpoIIGB	P06222
22	NtrC (Brady.)	P10576
23	DicA	P06966
24	SpoIIIC	P12254
25	EbgR	P06846
26	Lambda cII	P03042
27	Fis	P0A6R3
28	NifA	P03027
29	CytR	P0ACN7
30	PurR	P0ACP7
