# Supplementary material for: The Construction and Use of Log-Odds Substitution Scores for Multiple Sequence Alignment
Source: PLoS Comput Biol. 2010 Jul 15;6(7):e1000852. doi: 10.1371/journal.pcbi.1000852 (PMC2904766; doi:10.1371/journal.pcbi.1000852)
Supplement: Table S2 — Number of sequences misaligned by Gibbs sampling programs. Sequence sets supplied to the BILD and Wadsworth samplers consist of the first M sequences listed in Table S1. For each sequence set, the BILD sampler determines an optimal motif width W. Both BILD and Wadsworth samplers optimize contiguous motifs of widths W, 17, 21 and 25. The number of sequences misaligned by the Wadsworth sampler are given in the table without parentheses; the number misaligned by the BILD sampler within parentheses. (0.02 MB PDF) [file pcbi.1000852.s007.pdf]

Table S2. Number of sequences misaligned by Gibbs sampling programs.

Sequence set size M	Optimal W	Pattern width W	Pattern width 17	Pattern width 21	Pattern width 25
30	22	2		1	3
29	22	2		1	2
28	22	2		1	3
27	22	1		1	2
26	22	1		1	2
25	22	1		1	1
24	22	1		1	2
23	22	1		1	2
22	22	1		1	2
21	22	1		1	2
20	22	1		1	2
19	20	1	3	1	2
18	20	1	3	1	2
17	20	1	3	1	2
16	20	1	3	1	4
15	20	1		1	4
14	20		(2)		1 (2)
13	20		(2)		
12	20		(2)		2
11	20		2		3
10	18	(1)	1 (2)	1	2 (2)
9	19	2		1	2 (1)
8	19				2
7	19			2	2
6	19	1 (1)	2 (1)	2	2 (1)
5	20	1 (1)	1 (1)	1 (1)	1 (1)
4	16	1 (1)	1 (1)	1 (1)	1 (1)
